# Synthesis, design, biological evaluation, and computational analysis of some novel uracil-azole derivatives as cytotoxic agents

**DOI:** 10.1186/s13065-023-01106-x

**Published:** 2024-01-03

**Authors:** Leila Emami, Fateme Zare, Soghra Khabnadideh, Zahra Rezaei, Zahra Sabahi, Saman Zare Gheshlaghi, Marzieh Behrouz, Mina Emami, Zahra Ghobadi, Sedighe Madadelahi Ardekani, Fatemeh Barzegar, Ali Ebrahimi, Razieh Sabet

**Affiliations:** 1 Pharmaceutical Sciences Research Center, Shiraz University of Medical Sciences, Shiraz, Iran; 2 Department of Medicinal Chemistry, Faculty of Pharmacy, Shiraz University of Medical Sciences, Shiraz, I.R. of Iran; 3 Department of Chemistry, Computational Quantum Chemistry Laboratory, University of Sistan and Baluchestan, Zahedan, Iran; 4 Department of Chemistry, Shiraz University of Technology, Shiraz, Iran

**Keywords:** Uracil-azole hybrids, Molecular dynamics simulation, ADME, DFT

## Abstract

The design and synthesis of novel cytotoxic agents is still an interesting topic for medicinal chemistry researchers due to the unwanted side effects of anticancer drugs. In this study, a novel series of uracil–azole hybrids were designed and synthesized. The cytotoxic activity, along with computational studies: molecular docking, molecular dynamic simulation, density functional theory, and ADME properties were also, evaluated. The compounds were synthesized by using 3-methyl-6-chlorouracil as the starting material. Cytotoxicity was determined using MTT assay in the breast carcinoma cell line (MCF-7) and Hepatocellular carcinoma cell line (HEPG-2). These derivatives demonstrated powerful inhibitory activity against breast and hepatocellular carcinoma cell lines in comparison to Cisplatin as positive control. Among these compounds, **4j** displayed the best selectivity profile and good activity with IC_50_ values of 16.18 ± 1.02 and 7.56 ± 5.28 µM against MCF-7 and HEPG-2 cell lines respectively. Structure–activity relationships revealed that the variation in the cytotoxic potency of the synthesized compounds was affected by various substitutions of benzyl moiety. The docking output showed that **4j** bind well in the active site of EGFR and formed a stable complex with the EGFR protein. DFT was used to investigate the reactivity descriptors of **4a** and **4j**. The outputs demonstrated that these uracil–azole hybrids can be considered as potential cytotoxic agents.

**Supplementary Information:**

The online version contains supplementary material available at 10.1186/s13065-023-01106-x.

## Introduction

Cancer is known as the second factor of mortality worldwide. Cancer arises from uncontrollable cell division due to mutation in genes [1]. The mutation occurred as a result of errors during mitosis or DNA damage generated by environmental or hereditary factors [2]. Complexity and heterogeneity of cancer, multi-drug resistance (MDR), as well as the undesired and intolerable side effects of chemotherapy, were the challenges faced in the treatment of this disease. Epidermal growth factor receptor (EGFR) was a cell membrane receptor. Overexpression and mutation of EGFR are the main factors in many types of cancers [3]. The clinical drugs related to EGFR act as tyrosine kinase inhibitors (Erlotinib, Gefitinib) [4] or as monoclonal antibodies (Necitumumab, Cetuximab) [5].

Most anticancer drugs bear heterocyclic scaffolds in their structure. Pyrimidine is a 6-membered heterocyclic ring, also known as 1,3-diazine which is naturally presented in the structure of purines, nucleotides, and nucleic acids [6]. The existence of this scaffold in the structure of DNA and RNA has caused attention to the design of containing anticancer drugs such as methotrexate, cytarabine, and 5-fluorouracil [7]. Recently, molecular hybridization has been used for the rational design of new compounds, in which two or more fragments were fused [8]. Usually, these fragments were selected according to the structure of existing drugs or bioactive compounds synthesized in previous studies [9, 10]. In the design of cytotoxic compounds, the types of heterocycle motives have been used [8, 11]. These derivatives were capable of inhibiting the key enzymes of the biosynthesis pathway of nucleotides. Uracil is a pyrimidine-2,4-dione that its hybrids with imidazole [12], oxadiazole [13], indole [14], coumarin [15], etc. have been reported as bioactive agents. Hybridization of uracil as a small molecule having an anti-cancer role with other effective heterocycles can help in designing potent compounds [16–18].

In the present study, we synthesized some hybrids of uracil with triazole and imidazole rings. These scaffolds can form hydrogen bonds through N-H, which improves the pharmacokinetic properties by increasing the solubility of the ligand [18, 19]. Also, based on reported studies, imidazole is known as a tubulin modulator and can overcome drug resistance [20, 21]. Our previous studies have shown that azole rings act as bioactive pharmacophores due to their structural features [22, 23]. The aim of the current study is to synthesize a series of derivatives containing uracil and azole with cytotoxic potential. Subsequently, the pharmacokinetic properties, docking studies, and molecular dynamics simulations have also been investigated for the synthesized compounds.

## Material and methods

### Reagents and solvents

All the starting materials, solvents, and reagents were procured from Merck Company (Germany). The melting points were measured with Electrothermal 9200 apparatus (Electrothermal, UK). Each compound’s structure was confirmed by Infrared spectra (VERTEX70 spectrometer,), ^1^H NMR and ^13^C NMR spectra (500 MHz, VARIAN-INOVA Bruker spectrophotometer in deuterated CDCl_3_ solution). The chemical shifts (δ) were reported in parts per million (ppm). The Mass spectra were recorded using Agilent Technologies, USA (70 e. v.).

### Synthesis

#### Synthesis of benzylated or benzoylated 3-methyl 6-chloro uracil (compounds 2 and 3)

Firstly, 1 mmol of benzyl bromide derivatives or benzoyl chloride were poured into a 100 ml round-bottomed flask, then 1 mmol of 3- methyl-6-chlorouracil and 2 mmol of diisopropylethylamine (DIPEA) were added to the reaction medium and the mixture was stirred at 40 °C for 10 min. The completion of the reaction was determined by TLC. After evaporation of the solvent, intermediates **2** and **3** were obtained.

#### Synthesis of 3-methyl-1-(substituted benzyl or benzoyl)-6-(azole substituted-1-yl) pyrimidine-2,4(1H,3H)-dione derivatives (4a-4 l)

Firstly, 1 mmol of the intermediate **2** or **3** was added to 1 mmol of various azole derivatives in the presence of 1 mmol of potassium carbonate and triethylamine in equal proportions and were refluxed at 75 °C for 24 h in acetonitrile as solvent. The progress of the reaction was controlled by TLC. The solvent was evaporated by rotary and extraction was done with ethyl acetate solvent. The organic part was collected and dehydrated with sodium sulfate, after filtration, the solvent was evaporated by rotary. Finally, the purification was conducted by plate chromatography. The final products of **(4a-4l)** were confirmed using ^1^HNMR, ^13^CNMR, MASS, and IR spectroscopic methods.

#### Spectra data

**Synthesis of 3-methyl-1-(benzyl)-6- (H**_**1**_**-imidazole-1-yl) pyrimidine- 2,4-(H**_**1**_**, H**_**3**_**) dione (4a):** Yield: 55%; m.p. 138–142 ºC**,** MS m/z (%): 91.2 (100), 65.1 (22.6), 158.1 (9.9), 43.1 (9.3), 282.1 (3.8). ^1^H-NMR (400 MHz, CDCl_3_) δ (ppm) = 7.3 (s, 1H, imidazole), 7.21–7.22 (m, 3H, phenyl), 7.12 (s, 1H, imidazole), 6.84–6.86 (m, 3H, 2H-phenyl + 1H-imidazole), 5.73 (s, 1H, uracil), 4.86 (s, 2H, CH_2_), 3.38 (s, 3H, CH_3_). ^13^C-NMR (100 MHz, CDCl_3_) δ (ppm) = 161.41, 151.75, 145.32, 137.01, 135.23, 131.09, 129.05, 128.37, 126.6, 119.45, 100.61, 48.18, 28.69. IR (KBr) v (cm^−1^): 1633–1662 (C=O), 1513 (C=N), 1448 (C=C), 1345–1379 (C-O), 1214–1286 (C-N).

**Synthesis of 3-methyl-1-(3-methyl benzyl)-6- (H**_**1**_**-imidazole-1-yl) pyrimidine- 2,4-(H**_**1**_**, H**_**3**_**) dione (4b):** Yield: 69%; m.p. 123–124 ºC; MS m/z (%): 296.1 (74.8), 205.1 (2.7), 171.1 (5.6), 132.1 (4.1), 105.1 (100), 77.1 (16.1), 52.1 (2.6). ^1^H-NMR (500 MHz, CDCl_3_) δ (ppm) = 7.37 (s, 1H, H_2_-imidazole), 7.19 (s, 1H, H_5_-imidazole), 7.15–7.16 (m, 1H, phenyl), 7.07–7.09 (m, 1H, phenyl), 6.91 (s, 1H, H_4_-imidazole), 6.69–6.71 (m, 2H, phenyl), 4.9 (s, 2H, CH_2_), 3.45 (s, 3H, CH_3_-uracil), 2.29 (s, 3H, CH_3_-aromatic). ^13^C-NMR (125 MHz, CDCl_3_) δ (ppm) = 161.44, 151.73, 145.38, 138.85, 137.02, 135.14, 130.97, 129.10, 128.90, 127.24, 123.52, 119.48, 100.54, 48.17, 28.67, 21.36. IR (KBr) v (cm^−1^): 1703–1672 (C=O), 1636 (C=N), 1454 (C=C), 1281 (C-N).

**Synthesis of 3-methyl-1-(4-methyl benzyl)-6- (H**_**1**_**-imidazole-1-yl) pyrimidine- 2,4-(H**_**1**_**, H**_**3**_**) dione (4c):** Yield: 69%; m.p. 100–102 ºC; MS m/z (%): 105.2 (100), 296.1 (64.9), 77.1 (24), 132.1 (7), 171.1 (6.8).^1^H-NMR (400 MHz, CDCl_3_) δ (ppm) = 7.3 (s, 1H, imidazole), 7.13 (s, 1H, imidazole), 7.01 (d, 2H, j = 8 Hz, Phenyl), 6.9 (s, 1H, imidazole), 6.73 (d, 2H, j = 8 Hz, Phenyl), 5.71 (s, 1H, uracil), 4.82 (s, 2H, CH_2_), 3.37 (s, 3H, CH_3_-uracil), 2.24 (s, 3H, CH_3_-phenyl). ^13^C-NMR (100 MHz, CDCl_3_) δ (ppm) = 161.45, 151.76, 145.38, 138.23, 137.07, 132.2, 131.11, 129.68, 126.65, 119.46, 100.51, 48.02, 28.67, 21.13. IR (KBr) v(cm^−1^): 1627–1667 (C=O), 1514–1534 (C=N), 1410–1453 (C=C), 1314–1377 (C-O), 1208–1294 (C-N).

**Synthesis of 3-methyl-1-(4-bromo benzyl)-6- (H**_**1**_**-imidazole-1-yl) pyrimidine- 2,4-(H**_**1**_**, H**_**3**_**) dione (4d**): Yield: 72%; m.p. 110–113 ºC; MS m/z (%):376.1 (56.4), 250.1 (4.8), 205.1 (8.6), 169.1 (100), 90.1 (38.3). ^1^H-NMR (500 MHz, CDCl_3_) δ (ppm) = 7.385 (d, 2H, j = 5 Hz, Phenyl), 7.10 (s, 1H, 2Methyl-Imidazol), 6.83 (s, 1H, 2Methyl-Imidazol), 6.76 (d, 2H, j = 10 Hz, Phenyl), 5.74 (s, 1H, uracil), 5.05 (d, 1H, j = 15 Hz, CH_2_), 4.54 (d, 1H, j = 15 Hz, CH_2_), 3.45 (s, 3H, CH_3_-uracil), 1.9 (s, 3H, CH_3_-2Methyl-Imidazol). ^13^C-NMR (125 MHz, CDCl_3_) δ (ppm) = 161.31, 151.91, 145.36, 145.25, 134.07, 132.16, 132.02, 129.93, 129.14, 128.29, 122.67, 119.25, 102.12, 47.14, 28.73, 12.65.

**Synthesis of 3-methyl- 1- (benzyl) 6- (2-methyl-H**_**1**_**-imidazol-1-yl) pyrimidine- 2,4- (H**_**1**_**, H**_**3**_**) dione (4e):** Yield: 55%; m.p. 138–140 ºC; MS m/z (%): 296.1 (85.6), 254.1 (8.6), 205.1 (6.1), 171.1 (8.4), 117.1 (12.6), 91.1 (100), 65.1 (25.8); ^1^H-NMR (500 MHz, CDCl_3_) δ (ppm) = 7.25–7.27 (m, 3H, phenyl), 7.09 (s, 1H, Imidazole), 6.85–6.87 (m, 3H, 2H-phenyl + 1H-Imidazole), 5.72 (s, 1H, uracil), 5.20 (d, 1H, j = 7.5 Hz, CH_2_), 4.55 (d, 1H, j = 15 Hz, CH_2_), 3.47 (s, 3H, CH_3_-uracil), 1.81 (s, 3H, CH_3_-2-Me-imidazole); ^**13**^**C-**NMR (125 MHz, CDCl_3_) δ (ppm)** = **161.50, 145.55, 135.15, 129.76, 128.88, 128.57, 127.29, 127.27, 119.21, 119.20, 101.99, 47.77, 28.71, 12.45; IR (KBr) v (cm^−1^): 1703–1672 (C=O), 1632 (C=N), 1450 (C=C), 1287 (C-O), 1178 (C-N).

**Synthesis of 3-methyl-1-(4-methyl benzyl)-6- (2-methyl-H**_**1**_**-imidazole-1-yl) pyrimidine- 2,4-(H1, H3) dione (4f)**: Yield: 58%; m.p. 120–122 ºC; MS m/z (%): 310.2 (91.8), 268.1 (5.2), 205.1 (5.6), 105.1 (100), 77.1 (21.6). ^1^H-NMR (500 MHz, CDCl_3_) δ (ppm) = 7.09 (s, 1H, 2Methyl-Imidazol), 7.05 (d, 2H, j = 10 Hz, Phenyl), 6.85 (s, 1H, 2Methyl-Imidazol), 6.75 (d, 2H, j = 10 Hz, Phenyl), 5.72 (s, 1H, uracil), 5.15 (d, 1H, j = 15 Hz, CH_2_), 4.52 (d, 1H, j = 15 Hz, CH_2_), 3.48 (s, 3H, CH_3_-uracil), 2.29 (s, 3H, CH_3_-2-Methyl-Imidazol), 1.84 (s, 3H, CH_3_- Phenyl). ^13^C-NMR (125 MHz, CDCl_3_) δ (ppm) = 161.55, 152.01, 145.62, 145.51, 138.41, 132.11, 129.74, 129.48, 127.29, 1119.23, 101.88, 47.60, 28.69, 21.08, 12.54. IR (KBr) v (cm^−1^): 1647–1630 (C=O), 1547 (C=N), 1483 (C=C), 1379 (C-O), 1273 (C-N), 1148(C-C).

**Synthesis of 3-methyl-1-(4-bromo benzyl)-6- (2-methyl-H**_**1**_**-imidazole-1-yl) pyrimidine- 2,4-(H**_**1**_**, H**_**3**_**) dione (4g)**: Yield: 68%; m.p. 160–162 ºC; MS m/z (%):376.1 (56.4), 250.1 (4.8), 205.1 (8.6), 169.1 (100), 90.1 (38.3). ^1^H-NMR (500 MHz, CDCl_3_) δ (ppm) = 7.385 (d, 2H, j = 5 Hz, Phenyl), 7.10 (s, 1H, 2Methyl-Imidazol), 6.83 (s, 1H, 2Methyl-Imidazol), 6.76 (d, 2H, j = 10 Hz, Phenyl), 5.74 (s, 1H, uracil), 5.05 (d, 1H, j = 15 Hz, CH_2_), 4.54 (d, 1H, j = 15 Hz, CH_2_), 3.45 (s, 3H, CH_3_-uracil), 1.9 (s, 3H, CH_3_-2Methyl-Imidazol). ^13^C-NMR (125 MHz, CDCl_3_) δ (ppm) = 161.31, 151.91, 145.36, 145.25, 134.07, 132.16, 132.02, 129.93, 129.14, 128.29, 122.67, 119.25, 102.12, 47.14, 28.73, 12.65 (Additional file 1).

**Synthesis of 3-methyl-1-(benzyl)-6- (H**_**1**_**-1,2,4-triazole-1-yl) pyrimidine- 2,4-(H**_**1**_**, H**_**3**_**) dione (3h):** Yield: 58%; m.p. 91–93 ºC; MS m/z (%): 297.1 (40.4), 143.1 (4.1), 105.2 (100), 77.1 (12.3), 51.1 (1.9). ^1^H-NMR (500 MHz, CDCl_3_) δ (ppm) = 8.16 (s, 1H, Triazole), 7.92 (s, 1H, Triazole), 7.28 (d, 2H, j = 10 Hz, Phenyl), 6.68 (d, 3H, j = 15 Hz, Phenyl), 5.75 (s, 1H, uracil), 3.38 (3H, CH_3_-uracil). ^13^C-NMR (125 MHz, CDCl_3_) δ (ppm) = 164.08, 161.05, 153.99, 151.52, 145.06, 144.02, 134.26, 132.09, 128.56, 122.45, 100.25, 47.03, 28.81. IR (KBr) v (cm^−1^): 1719-1703-1670 (C=O), 1535 (C=N), 1458 (C=C), 1281 (C-N).

**Synthesis of 3-methyl-1-(3-methylbenzyl)-6- (H**_**1**_**-1,2,4-triazole-1-yl) pyrimidine- 2,4-(H**_**1**_**, H**_**3**_**) dione (4i):** Yield: 72%; m.p. 125–128 ºC; MS m/z (%): 297.1 (65.8), 143.1 (8), 105.1 (100), 77.1 (2.8), 43.1 (9.1). ^1^H-NMR (500 MHz, CDCl_3_) δ (ppm) = 8.20 (s, 1H, Triazole), 7.85 (s, 1H, Triazole), 7.07–7.10 (m, 1H, phenyl), 7.02 (d, 1H, j = 5 Hz, phenyl), 6.58–6.62(m, 2H, phenyl), 5.80 (s, 1H, uracil), 5.16 (s, 2H, CH_2_), 3.45 (3H, CH_3_-uracil), 2.24 (3H, CH_3_- phenyl). ^13^C-NMR (125 MHz, CDCl_3_) δ (ppm) = 161.25, 153.771, 151.62, 145.00, 144.37, 138.74, 135.18, 129.07, 128.80, 127.29, 123.54, 100.30, 47.56, 21.29. IR (KBr) v (cm^−1^): 1703–1670 (C=O), 1535 (C=N), 1458 (C=C), 1348 (C-O), 1281 (C-N), 752 (C-C).

**Synthesis of 3-methyl-1-(4-methyl benzyl)-6- (H**_**1**_**-1,2,4-triazole-1-yl) pyrimidine- 2,4-(H**_**1**_**, H**_**3**_**) dione (4j)**: Yield: 68%; m.p. 116–118 ºC; MS m/z (%): 297.1 (65.8), 143.1 (8), 105.1 (100), 77.1 (2.8), 43.1 (9.1). ^1^H-NMR (500 MHz, CDCl_3_) δ (ppm) = 8.20 (s, 1H, Triazole), 7.85 (s, 1H, Triazole), 7.07–7.10 (m, 1H, phenyl), 7.02 (d, 1H, j = 5 Hz, phenyl), 6.58–6.62(m, 2H, phenyl), 5.80 (s, 1H, uracil), 5.16 (s, 2H, CH_2_), 3.45 (3H, CH_3_-uracil), 2.24 (3H, CH_3_- phenyl). ^13^C-NMR (125 MHz, CDCl_3_) δ (ppm) = 161.25, 153.771, 151.62, 145.00, 144.37, 138.74, 135.18, 129.07, 128.80, 127.29, 123.54, 100.30, 47.56, 21.29. IR (KBr) v (cm^−1^): 1703–1670 (C=O), 1535 (C=N), 1458 (C=C), 1348 (C-O), 1281 (C-N), 752 (C-C).

**Synthesis of 3-methyl-1-(4-bromo benzyl)-6- (H**_**1-**_**1,2,4-triazole-1-yl) pyrimidine- 2,4-(H**_**1**_**, H**_**3**_**) dione (4k)**. Yield: 85%; m.p. 122-125ºC; MS m/z (%): 169.1 (100), 90.1 (32.64), 361.1 (12.71), 63.1 (7.22), 128.1 (7.18). ^1^H-NMR (400 MHz, CDCl_3_) δ (ppm) = 8.16 (s, 1H, triazole), 7.92 (s, 1H, triazole), 7.28 (d, 2H, j = 8.4 Hz, phenyl), 6.69 (d, 2H, j = 8.4 Hz, phenyl), 5.75 (s, 1H, uracil), 5.08 (s, 2H, CH_2_), 3.38 (s, 3H, CH_3_). ^13^C-NMR (100 MHz, CDCl_3_) δ (ppm) = 161.05, 153.98, 151.52, 145.08, 144.02, 134.25, 132.09, 128.56, 122.45, 100.25, 47.03, 28.81. IR (KBr) v (cm^−1^): 1634–1657 (C=O), 1508–1537 (C=N), 1401–1478 (C=C), 1339–1372 (C-O), 1221–1280 (C-N).

**Synthesis of 3-methyl-1-( phenyl)-6- (H**_**1**_**-1,2,4-triazole-1-yl) pyrimidine- 2,4-(H**_**1**_**, H**_**3**_**) dione (4l):** Yield: 85%; m.p. 99-101ºC; MS m/z (%): 91.2 (100), 283.1 (49.4), 65.1 (28.4), 129.1 (16.2), 51.1 (7.71). ^1^H-NMR (400 MHz, CDCl_3_) δ (ppm) = 8.14 (s, 1H, triazole), 7.79 (s, 1H, triazole), 7.12–7.2 (m, 3H, Phenyl), 6.75–6.77 (m, 2H, Phenyl), 5.73 (s, 1H, uracil), 5.14 (s, 2H, CH_2_), 3.4 (s, 3H, CH_3_). ^13^C-NMR (100 MHz, CDCl_3_) δ (ppm) = 161.22, 153.81, 151.63, 145, 144.31, 135.26, 128.96, 128.35, 126.62, 100.31, 47.59, 28.82. IR (KBr) v (cm^−1^): 1638–1661 (C=O), 1516 (C=N), 1422–1480 (C=C), 1346–1395 (C-O), 1238–1298 (C-N).

### MTT assay

The cytotoxic activity of the designed compounds **(4a-4l)** was obtained by MTT assay (standard 3-(4,5-dimethylthiazol-yl)-2,5-diphenyl-tetrazolium bromide) according to our previous protocols [24, 25]. Two human cancer cell lines including MCF-7 (breast carcinoma) and HEPG-2 (Hepatocellular carcinoma) were purchased from the National Cell Bank of Iran (NCBI, Pasteur Institute, Tehran, Iran). RPMI 1640 culture media was used to culture cancer cell lines. The media was supplemented with 10% fetal bovine serum (FBS) and 1% penicillin–streptomycin (Gibco, USA), and the cells were kept at 37 °C in a humidified CO_2_ incubator. For the MTT assay, trypsin/EDTA 0.5% solution (Gibco/USA) was applied to harvest cells and seeded in 96-well microplates at a density of 1 × 10^4^ cells per well [26]. Five different concentrations of the designed compounds and Cisplatin as the positive control (1–200 μM) were used for treatment in triplicate times. Three untreated wells were used as the negative control. After 72 h, the media was changed by 100 μL fresh MTT solution and incubated for 4 h at 37 °C in the incubator to obtain formazan purple crystals [27]. Finally, the media was removed and 150 μL of DMSO was added and incubated at 37 °C in dark for 10 min to dissolve the crystals. The absorbance of individual wells was read at 490 nm using a microplate ELISA reader. Excel 2016 and Curve Expert 1.4 were used to analyze the data. The data were presented as mean ± SD for each analysis.

### Molecular docking

Docking studies were conducted to understand the interaction and orientation of compounds with the active site of protein using AutoDock Vina. The crystal structure of EGFR was downloaded from the RCSB protein data bank site (PDB ID: 1M17) [28]. To prepare the compounds for the docking approach, the structures were minimized in terms of energy and converted to pdbqt format. A grid box of 30 × 30 × 30 Å and an exhaustiveness of 100 were set for docking analysis. The interaction and orientation of the compounds in the active site were visualized by Discovery Studio 2016.

### Molecular dynamic simulation

Molecular dynamics simulation (MD) was used for the observation of conformational changes in ligand–protein complex and evaluation of the accuracy of molecular docking results. The MD was run using GROMACS package 2020 during 100 ns and the AMBER99SB-LIDN force field. The partial charges were calculated with the AM1-BCC method using the Antechamber program of Amber Tools. The complexes were located in a cubic box with dimensions of 12 × 12 × 12 Å and dissolved in TIP3P water. The minimization of energy was performed using the steepest descent algorithm. The constant temperature of 300 K and the constant pressure of 1 bar were considered for NPT and NVT equilibrium, respectively. In the present study, the top-ranked two tested compounds **4a** and **4j** were simulated with the protein target of EGFR (PDB ID: 1M17). The stability of simulated systems was analyzed using root mean square deviation (RMSD), root mean square deviation (RMSF), radius of gyration (Rg) and number of hydrogen bonds. The MD trajectories were visualized by VMD.

### DFT analysis

Density functional theory was used to investigate the reactivity descriptors of **4a** and **4j** at the B3LYP/6–31 + G (d, p) level of theory. The molecular orbitals (HOMO and LUMO) and electrostatic surface potential energy were also studied in detail.

### ADME

The properties of absorption, distribution, metabolism and excretion (ADME), can be predicted the safety of studied compounds. ADME profiles were obtained using SWISSADME server.

## Results and discussion

### Design approach

**Fig. 1:**
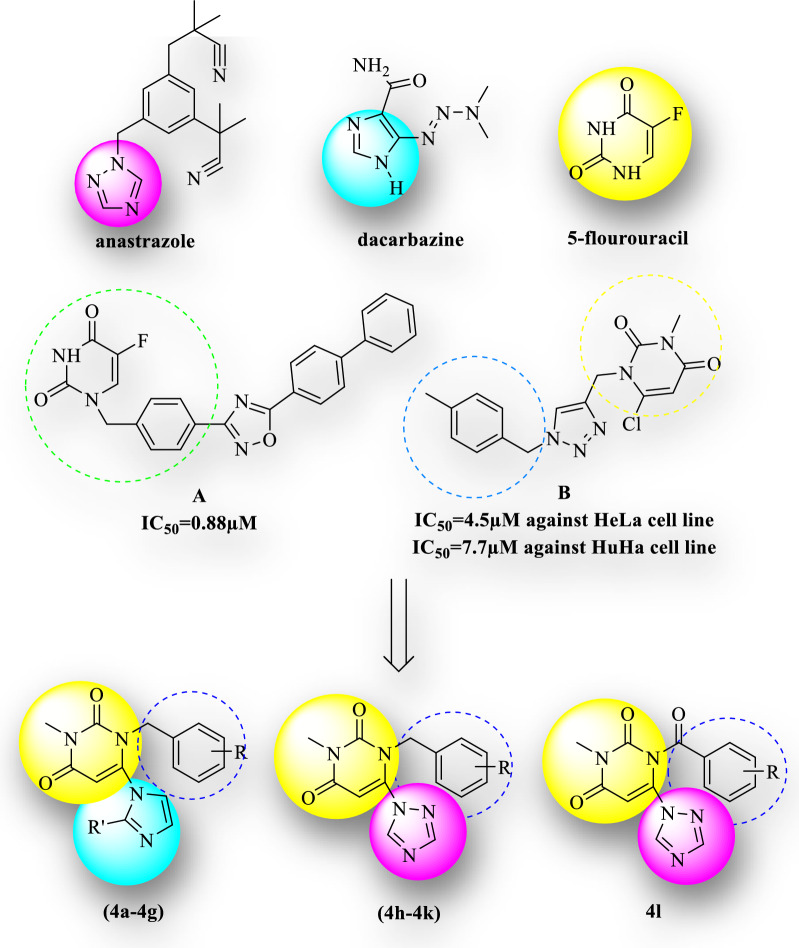
Design approach of the synthetic compounds **(4a-4l)**

Given the significant biological potential of uracil and azole derivatives as cytotoxic agents, we pursued the hybridization strategy for developing some new cytotoxic agents. Figure 1 shows some of the drugs including uracil, imidazole, and 1,2,4-triazole rings. Imidazole scaffold can inhibit the formation of cell membrane components via the formation of bonds with DNA and proteins [29]. 1,2,3-triazole has also been applied as a bulking agent in the synthesis of cytotoxic compounds [30]. In previous studies, hybrids of uracil with heterocycle groups were reported as potent compounds for cytotoxic activity. For example, compound A is a hybrid of uracil and oxadiazole with IC_50_ = 0.88 μM against a studied cancer cell line [13], and compound B, bearing uracil and 1,2,3-triazole exhibited a promising cytotoxic with IC_50_ = 4.5 and 7.7 μM against Hela and Huha cell lines respectively [31]. According to the stated items, 3-methyl–pyrimidine-2,4-dione was conjugated with various azole rings bearing benzyl or benzoyl substitution at the N-1 position of uracil with different electronic profiles (Fig. 1).

### General procedure for the synthesis of compounds (4a-4l)

**Fig. 2:**
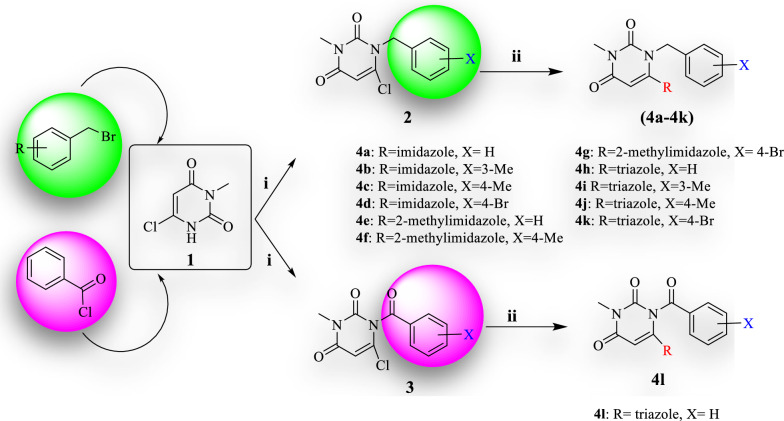
General schematic for the synthesis of compounds of **4a-4l**. Reagents and conditions: (i). Diisopropylethylamine (DIPEA), 40 ºC, 10 min; (ii) K_2_CO_3_, Azole moiety, TEA, Acetonitrile, 75 ºC, 24 h

In this study, some of the uracil-azole hybrid derivatives were synthesized in two steps. In the first step, 3-methyl-6-chlorouracil was reacted with various benzyl bromides and benzoyl chloride to produce the benzylated or benzoylated uracil intermediates **2** and **3**. Secondly, from the reaction between the intermediate substance **2** or **3** with azole derivatives including, imidazole, 2-methylimidazole, and 1,2,4-triazole, the final compounds **(4a-4l)** were obtained. The synthetic route of compounds is shown in Fig. 2.

### Biological activity

Twelve uracil-azole derivatives were designed and synthesized as cytotoxic agents. They represented appropriate activity in a range of 16.18–150.5 µM against MCF-7 and 7.56–193.3 µM against HEPG-2 cell lines (Table 1). Cisplatin is used as a standard drug, it is a reasonable standard cytotoxic agent that is a well-known chemotherapy medication, used to treat a wide range of cancers including; breast and Hepatocellular cancer, which their related cancerous cell lines were used for the MTT assay in this study. Compounds **4j**, **4b** and **4c** showed the best antiproliferative activity with IC_50_ values of 16.18, 17.12, and 26.13 µM against MCF-7 and 7.56, 48.47, and 27.18 µM against HEPG-2 cell lines, respectively. The compounds can be classified into three categories according to the azole substituent at C-5 of the uracil ring. Among the first-class bearing imidazole moiety (**4a**-**4d**), the introduction of electron donating group such as methyl at meta or para position of benzyl ring can improve activity in a range of 17.12–48.47 µM in **4b** and **4c** analogs. Replacement of the methyl group with electron withdrawing substituent (Br) in **4d** led to decrease activity (7–8 folds) compared to **4b** and **4c**. On the other hand, **4a** as un-substituted derivative cannot improve activity, either. In the case of the second class, **4e**-**4g** with 2 methyl imidazole motifs, it can be noticed that, the least potency belongs to this category in a range of 55.85–109.2 µM compared to other derivatives.

**Table 1:** The antiproliferative activities of the designed compounds **(4a-4l)**
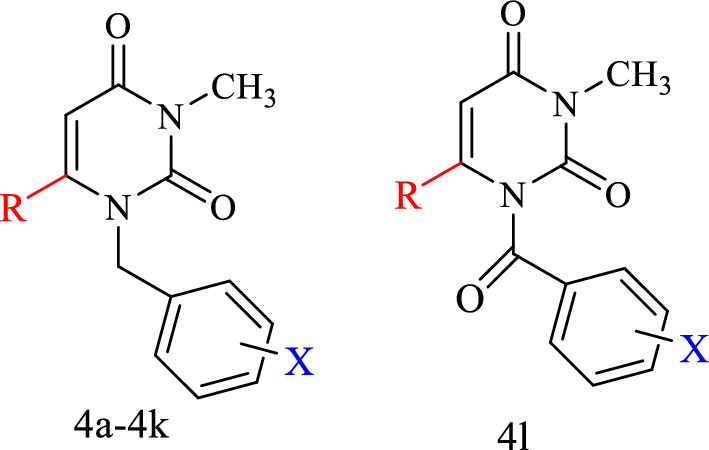

IC_50_ ± SD (µM)						IC_50_ ± SD (µM)					
Entry	R	X				Entry	R	X			
			MCF-7	HEPG-2	MRC-5				MCF-7	HEPG-2	MRC-5
**4a**	Imidazole	H	150.5 ± 0.77	193.3 ± 7.98	> 200	**4g**	2-Me-Imidazole	4-Br	109.2 ± 3.74	105.65 ± 3.03	> 200
**4b**	Imidazole	3-Me	17.12 ± 1.30	48.47 ± 1.97	61.8 ± 2.30	**4h**	Triazole	H	131.4 ± 2.14	108.15 ± 10.81	> 200
**4c**	Imidazole	4-Me	26.13 ± 4.41	27.18 ± 1.66	101.3 ± 3.02	**4i**	Triazole	3-Me	43.1 ± 2.07	65.40 ± 2.15	> 200
**4d**	Imidazole	4-Br	130.63 ± 3.1	177.4 ± 4.06	> 200	**4j**	Triazole	4-Me	16.18 ± 1.02	7.56 ± 5.28	57.3 ± 2.08
**4e**	2-Me-Imidazole	H	91.95 ± 2.19	ND	> 200	**4k**	Triazole	4-Br	78.15 ± 4.64	56.42 ± 2.46	> 200
**4f**	2-Me-Imidazole	4-Me	65.45 ± 3.1	55.85 ± 6.58	> 200	**4l**	Triazole	H	67.55 ± 2.41	72.8 ± 2.19	> 200
**Cis platin**		–	20.70 ± 0.83	15.91 ± 1.83	45.2 ± 2.5						

The number of compounds are shown with bold values **(4a-4l)**

*ND* Not Determined

**Fig. 3:**
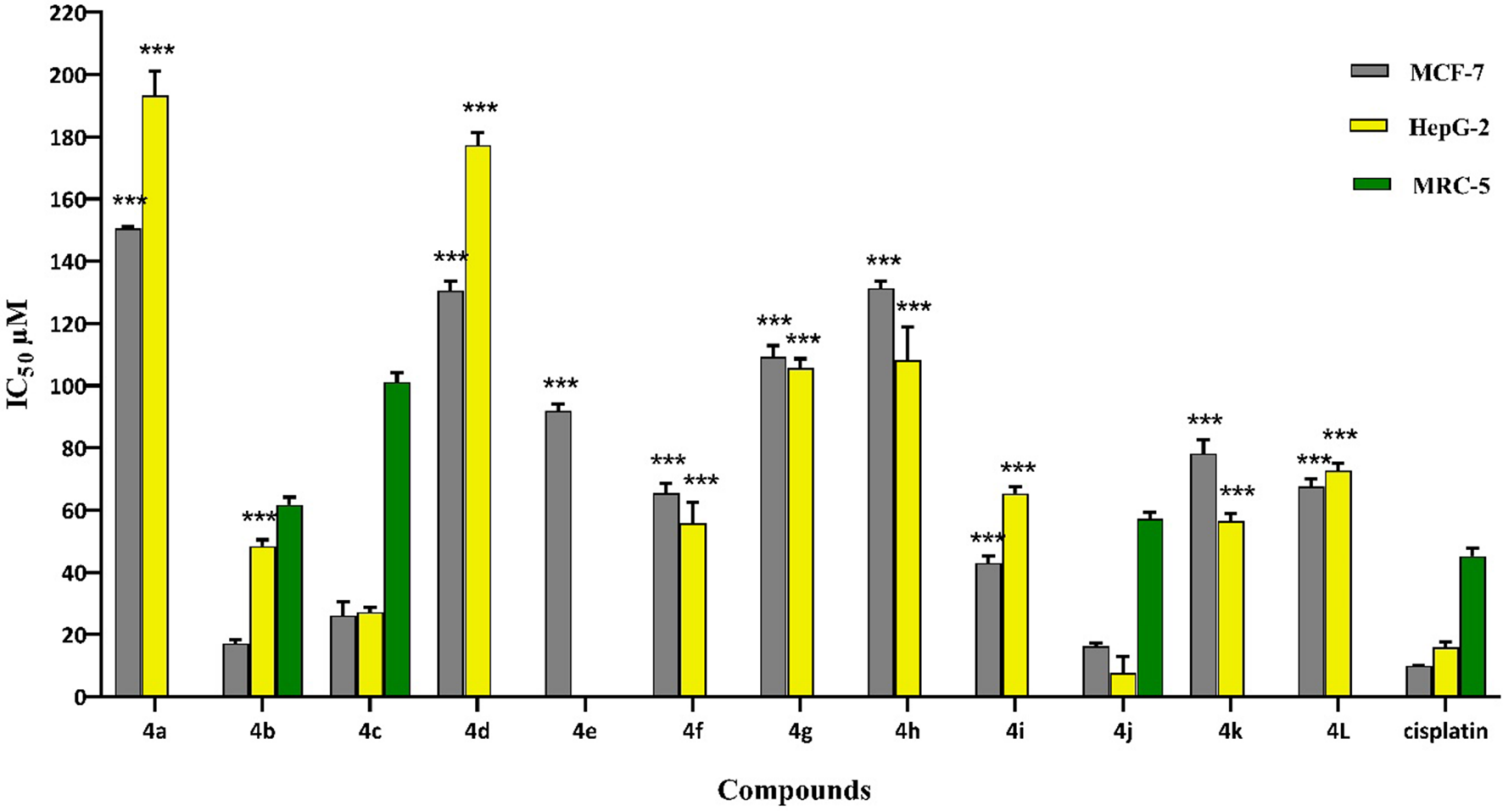
Cytotoxic effects of compounds **(4a-4l)** on MCF-7 and HEPG-2 cell lines. ∗ , ∗  ∗ , and ∗  ∗  ∗ indicate p < 0:05, p < 0:01, and p < 0:001 respectively compared to Cisplatin

Category 3 has five analogs (**4h**-**4l**) with triazole moiety showed a promising potency in a range of 7.56–131.4 µM. Compound **4j** bearing methyl group at the para position of benzyl moiety demonstrated good potency against the two studied cell lines. In this series, similar to the first category, electron donating substitutions improved the antiproliferative activity. 4-bromo and benzoyl substitutions as electron withdrawing groups determined a decrement in the potency compared to **4j**. Compound **4h** as un-substituted analogue like other categories had the least activity toward MCF-7 and HEPG-2 cell lines. Structure–activity relationship revealed that compounds containing triazole and imidazole moiety with electron donating substituent had the most potency. Assessment of the electron-donating group, placing at para position of benzyl moiety is more effective compared to meta counterpart. As shown in Fig. 3, compounds **4f**-**4h** and **4j**-**4k** are more sensitive against HEPG-2 compared to the MCF-7 cell line. Taken together, analogs **4j** represented remarkable potential in MCF-7 and HEPG-2 cell lines and **4b** had appropriate activity in the MCF-7 cell line compared to Cisplatin as positive control. As shown in Table 1, all of the compounds had a low cytotoxic potential on the normal lung cell line (MRC-5) compared to MCF-7 and HEPG-2 cancer cell lines which revealed that all studied synthesized compounds represented a desire selectivity between normal and cancerous cell lines.

### Molecular docking study

**Fig. 4:**
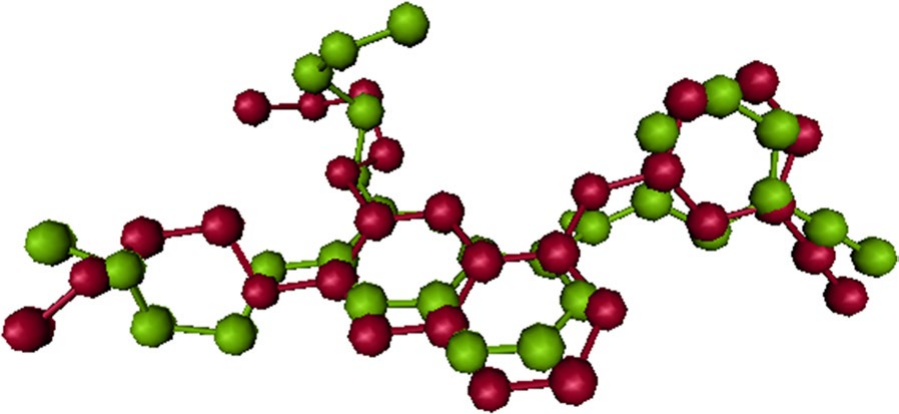
Two different conformations of Erlotinib in the active site of EGFR (PDB: 1M17): a red color indicated the reduced model and a green color illustrated the crystal orientation

Molecular docking was conducted to study the interaction and placement of the tested compounds in the binding site of the EGFR target as a plausible mechanism [32–36]. Co-crystal ligand (Erlotinib) was exactly placed in the active site of the receptor, redocking of the co-crystal ligand was done, and RMSD of docking was found to be 1.78. It indicates the validity of the docking process. The results are presented in Fig. 4.

**Fig. 5:**
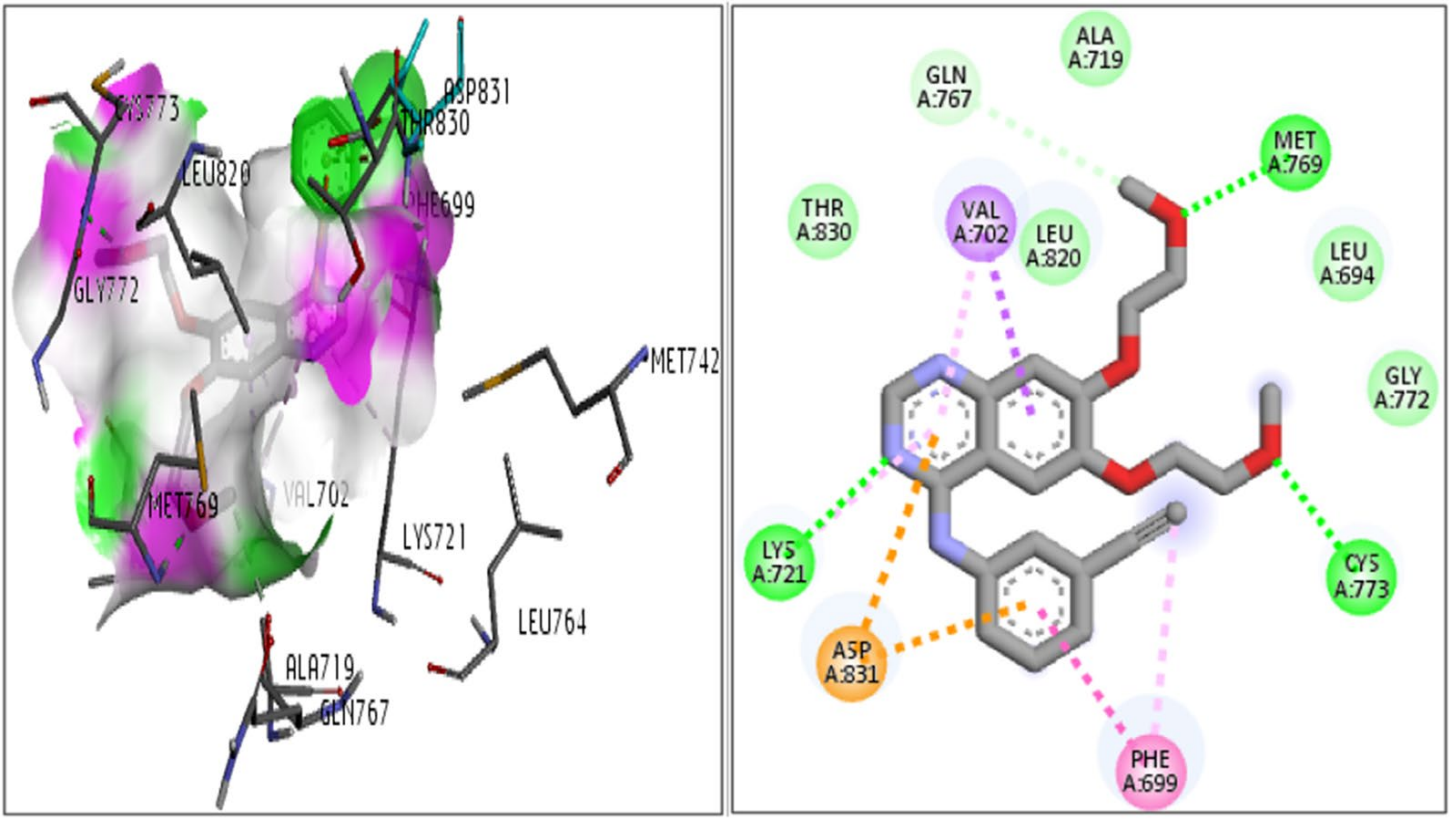
Interaction and orientation of Erlotinib in the active site of 1M17

The types of interaction and binding pose of Erlotinib as internal ligand in the active site of 1M17 protein are shown in Fig. 5. The nitrogen atom of the uracil ring and the oxygen atoms of the ethers formed hydrogen bond interactions with the residues of Lys721, Cys733, Met769. Other important interactions of this compound are π-sigma, π-stacking and π-anion bonds of six-membered rings with Val702, Phe699, and Asp831 residues. The docking score for Erlotinib in active site of 1M17 was obtained at -7.8 Kcal/mol. The docking score, and the critical interactions (hydrogen bonding and π contacts) for all the synthesized compounds are listed in Table 2. As in Table 2 is evident, the studied compounds had docking scores in the range of − 7.2 to − 8.5 Kcalmol^-1^, except **4a**, **4d**, **4e, 4g** and **4h,** the rest of the compounds had a higher docking score than Erlotinib. In addition, the compounds **4b**, **4c, 4f, 4i,** and **4j** had the highest docking score.

**Table 2:** Docking scores, critical interactions of the synthesized compounds (4a-4 l)

ID	Docking score (Kcal/mol)	π interaction	Hydrogen bonding (Distance Å)
4a	− 7.5	Lys721, Met742	–
4b	− 8.3	Asp831, Cys751, Val702	Met769 (2.89)
4c	− 8.5	Phe699, Asp831, Cys751, Met742	Met769 (3.09)
4d	− 7.2	–	–
4e	− 7.7	Lys721, Met742	–
4f	− 8.2	Phe699, Asp831, Met742	Met769 (2.96)
4g	− 7.4	Phe699	–
4h	− 7.5	Met742	–
4i	− 8.0	Met742, Lys721	–
4j	− 8.5	Leu820, Asp831	Lys721 (3.02) Thr830 (3.23)
4k	− 7.9	Asp831	Lys721 (3.01)
4l	− 7.8	Asp831, Leu820, Phe699	–
Erlotinib	− 7.8	Asp831, Val702, Phe699	Met769, Lys721, Cys773

**Fig. 6:**
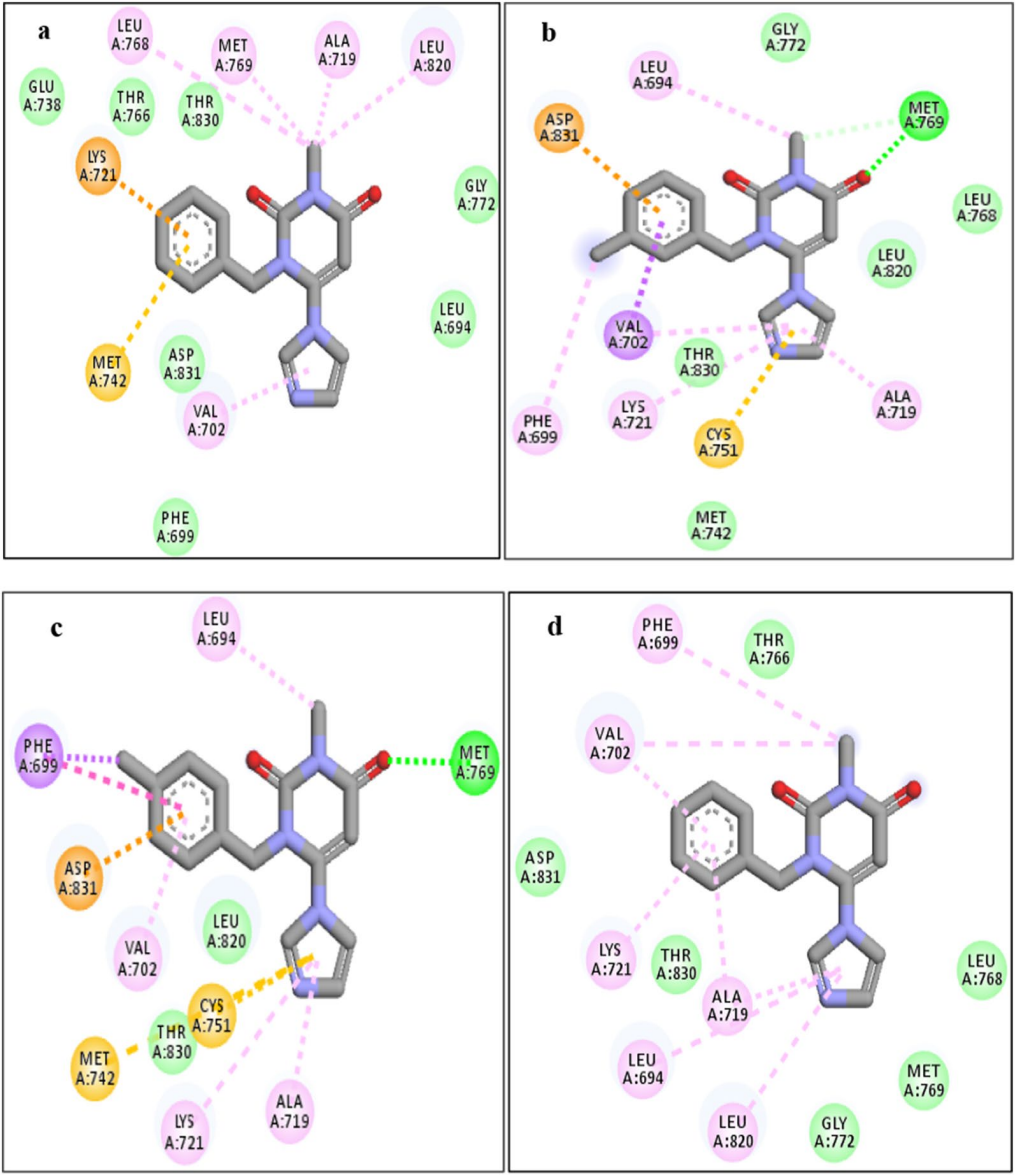
Interaction and orientation of the synthesized compounds of **(4a-4d)**

**Fig. 7:**
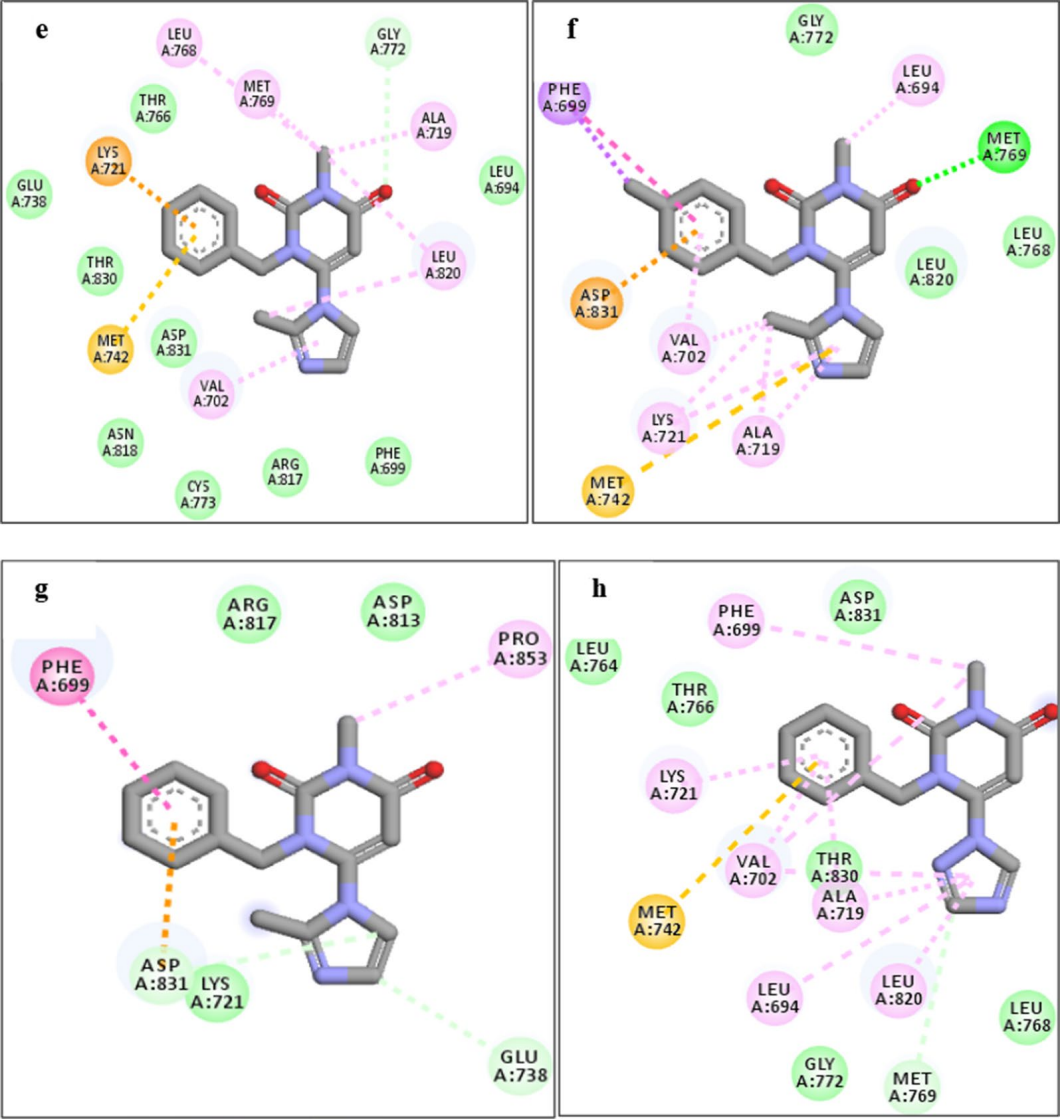
Interaction and orientation of the synthesized compounds of **(4e-4h)**

**Fig. 8:**
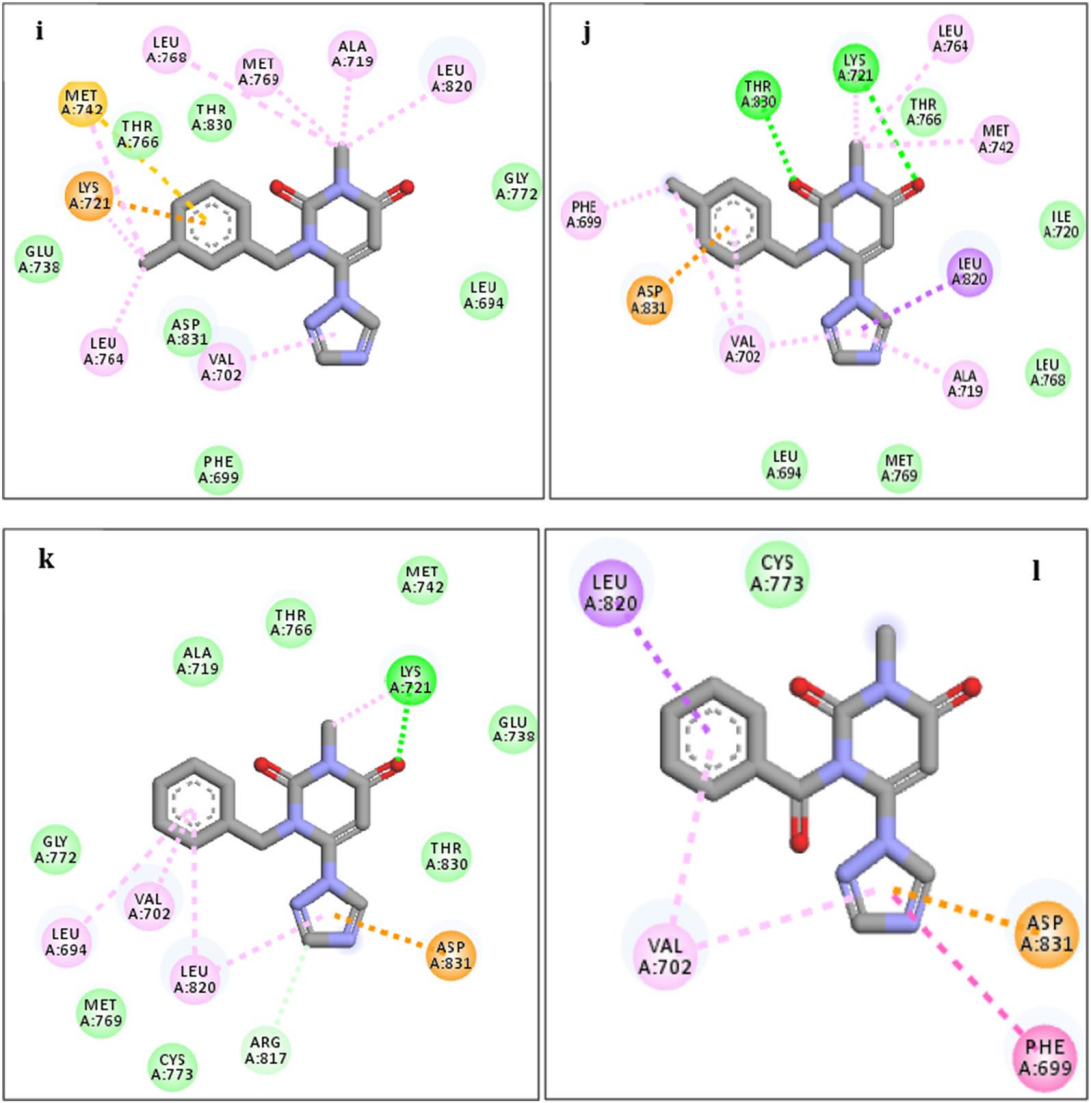
Interaction and orientation of the synthesized compounds of **(4i-4l)**

The analysis of interference and orientation of some studied compounds in the active site of 1M17 are shown in Figs. 6, 7, 8. As can be observed, all the studied compounds were located in the binding pocket of the EGFR enzyme. Among the tested derivatives, compounds **4c** and **4j** showed the highest docking score and contacted with key residues in the active site of the EGFR target. The compound **4c** interacted with key residues of Asp831, Phe699, and Met769, and compound **4j** established the interactions with key residues of Lys721 and Asp831. Obtained results predicted that the compounds of **4b, 4c, 4f,** and **4j** contacted tightly with the binding site EGFR protein which confirmed the cytotoxic potential. Also, the docking outputs for less active compounds for example **4a, 4d, 4e, 4g,** and **4h** were according to their biological results.

### Molecular dynamics simulation

**Fig. 9:**
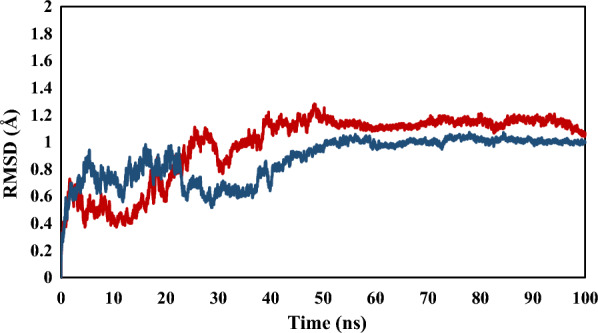
The results of RMSD analysis for selected compounds of **4a** (red) and **4j** (blue) in simulation time of 100

Based on docking results, compounds **4a** and **4j** were analyzed for molecular dynamics simulation during 100 ns. The RMSD analysis indicated the stability of the protein–ligand complex [37]. The results of RMSD analysis for **4a** and **4j** are given in Fig. 9. Both complexes **4a** and **4j** fluctuated in the first 45 ns of the simulation time, and after a period, the fluctuation graphs reached the plateau until the end of the simulation. These results revealed the stability of the protein–ligand complex for ligands **4a** and **4j** in the rest of the simulation time.

**Fig. 10:**
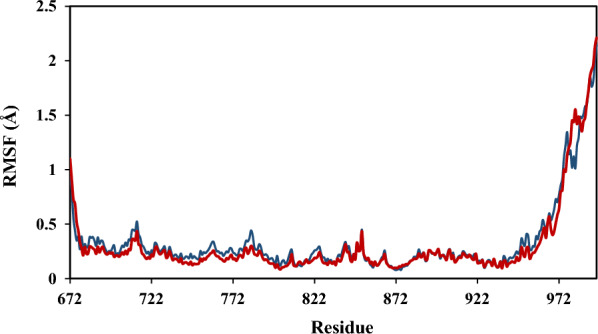
The results of RMSF analysis for selected compounds of **4a** (red) and **4j** (blue) in simulation time of 100

The RMSF analysis described the deviation of each amino acid residue and thus the stability of the protein–ligand complex [23]. The RMSF graphs for both complexes of **4a** and **4j** are presented in Fig. 10. The lower RMSF values indicated fewer fluctuations of amino acid residues. The obtained average RMSF values of 0.33 and 0.30 for **4a** and **4j**, respectively, suggested small fluctuations of amino acids for both complexes. Also, the results displayed fewer deviations for compound **4j** compared to compound **4a**. As expected, the key amino acids in the binding site had lower RMSF values thus our results confirmed small RMSF values for critical residues of Phe699, Val702, Lys721, Cys773, Met769, and Asp831.

**Fig. 11:**
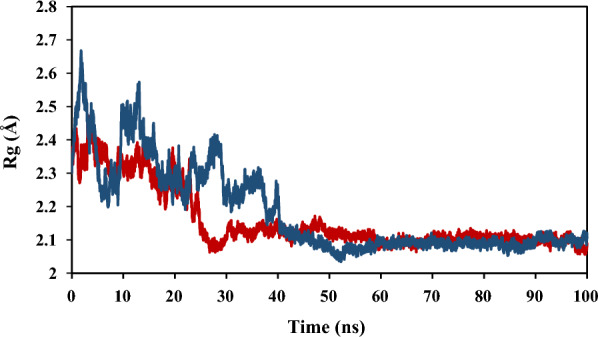
The results of Rg analysis for selected compounds of **4a** (red) and **4j** (blue) in simulation time of 100

The Rg analysis indicated the compactness of the protein–ligand complex [38], subsequently the lower the values of Rg, determined the stability of the ligand–protein complex. The results of Rg analysis are observed in Fig. 11. According to these results, the Rg values remained nearly constant after 40 ns from the beginning of the simulation time thereby demonstrated the rigidity and stability of both complexes.

**Fig. 12:**
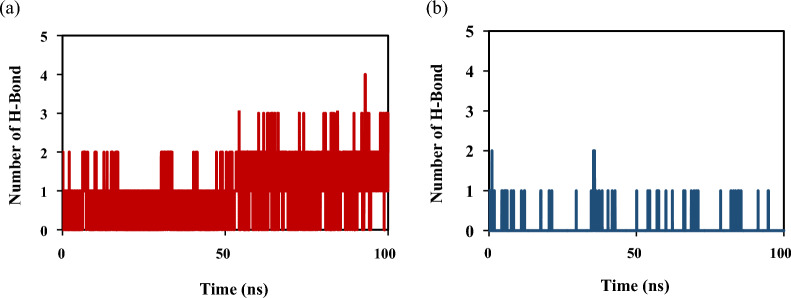
The number of hydrogen bonds of selected compounds of (a) **4a** and (b) **4j** in the simulation time of 100 ns

The number of hydrogen bonds was one of the main factors in the stability of the protein–ligand complex, because it was one of the important interactions in binding the ligand to the protein [39]. The formed number of hydrogen bonds for **4a** and **4j** in the simulation time is depicted in Fig. 12. The number of hydrogen bonds for compound **4a** varied between 0 and 3 and for compound **4j** between 0 and 1. The obtained results were consistent with docking results.

### DFT analysis

**Fig. 13:**
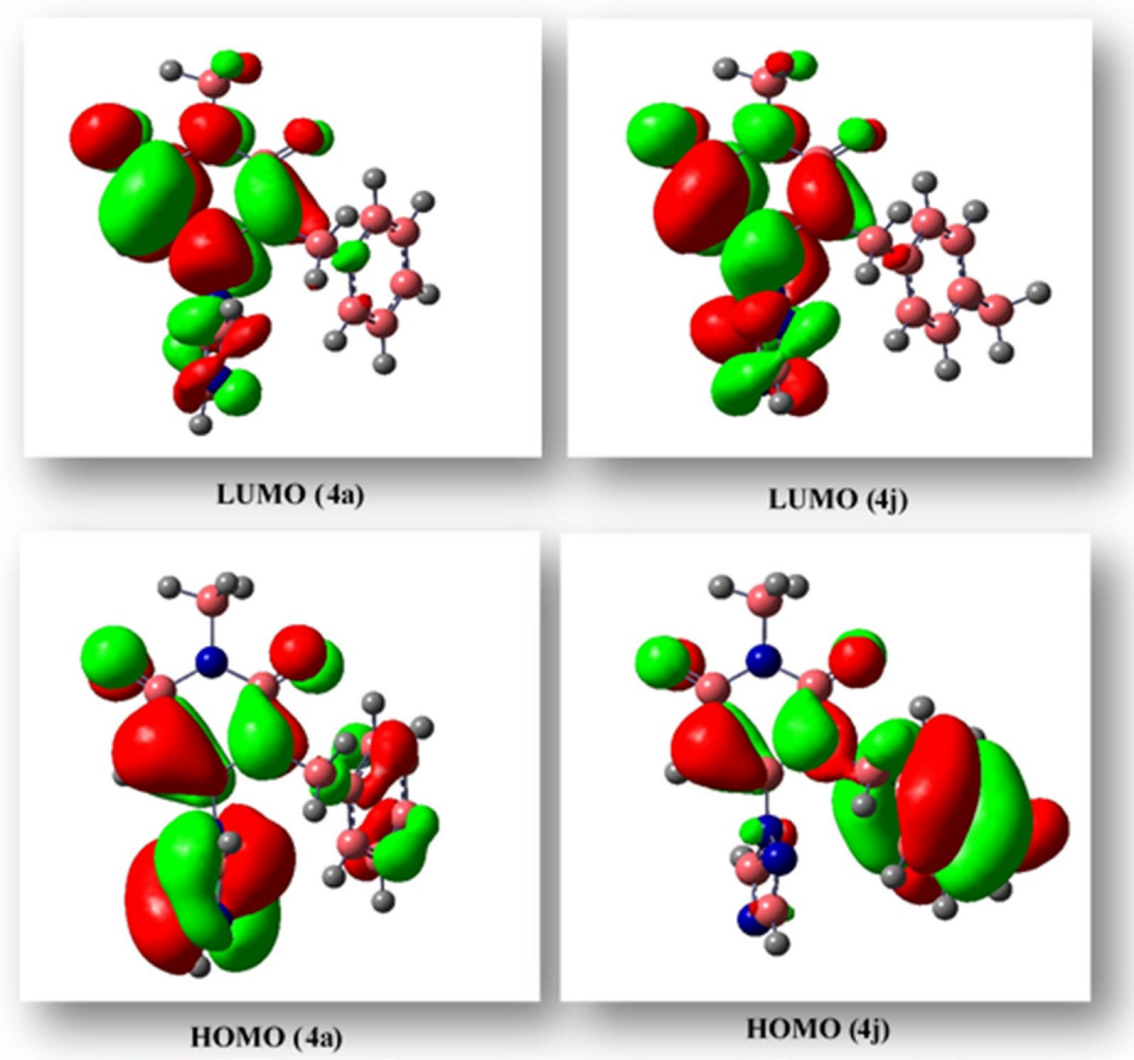
DFT calculated LUMO, HOMO, and their energies for **4a** (right) and **4j** (left) at the B3LYP/6–31 + G (d,p) level of theory

Geometry optimization without considering symmetry and CHELPG charge [40] for **4a** and **4j** was carried out with Gaussian 09 at the B3LYP/6–31 + G (d,p) level of theory. In addition, the frequency calculations ensured that there were no imaginary frequencies. The HOMO, LUMO, and their energy values are shown in Fig. 13. The location of the major part of HOMO in **4a** is on the imidazole motif while triazole is a negligible part of HOMO in **4j**. Furthermore, the phenyl ring has a significant part of HOMO in **4j**, and it is small in **4a**. The LUMO orbitals are not found on the phenyl ring in either molecule. The state of LUMO in both compounds exhibits similarity on the pink ring but displays dissimilarity on the imidazole (**4a**) and triazole (**4j**) rings. Also, there is a larger lobe in **4j** compared to **4a**. Furthermore, the lobes observed in the triazole ring of **4j** demonstrate a greater size in comparison to the imidazole ring in **4a**.

**Fig. 14:**
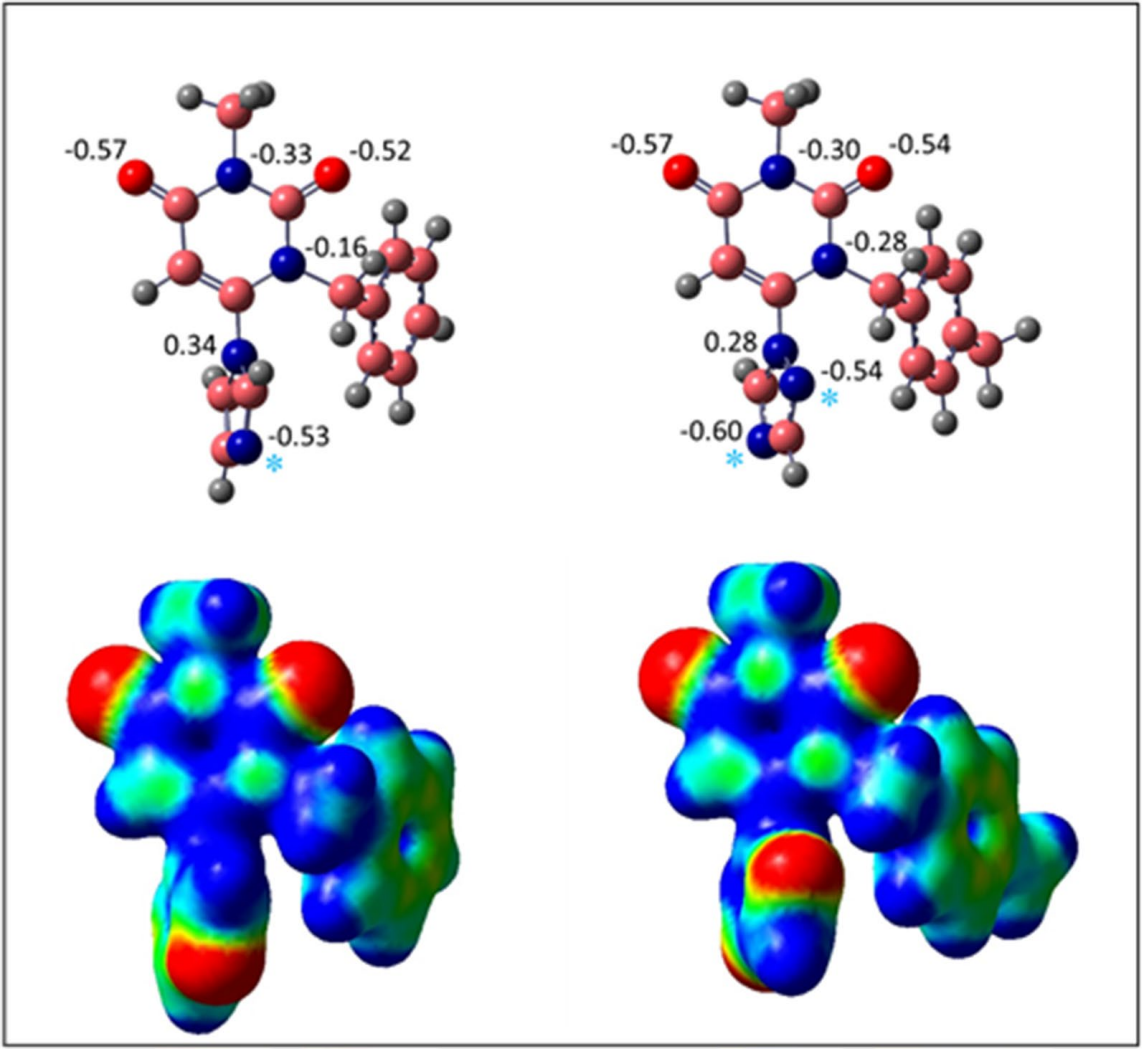
Geometry optimized and calculated CHELPG charge for hetero atoms as well as ESP of **4a** and **4j** at B3LYP/6–31 + G (d,p) level of theory

DFT-optimized structures of **4a** and **4j** were monitored in Fig. 14. The CHELPG charge of hetero atoms (O and N) in **4a** and **4j** were near atoms and in line with the electrostatic surface potential (ESP) energy of these compounds. The electron-rich parts were stated at the oxygen of the carbonyl group and in nitrogen atom in imidazole and triazole rings that were specified by cyan stars (*). These sites indicate the parts of molecules that are suitable for electrophilic reactions. The red spheres on the ESP graph represent the negative charge sites. According to the reactivity descriptors (Table 3), **4j** is more active than **4a**. Also, additional p-methyl on the phenyl ring increases the entropy of **4j** more than **4a**.

**Table 3:** The calculated total energy (E_tot_), Enthalpy (H), Gibbs free energy (G), hardness (ɳ), softness (σ), ionization energy (I) and electron affinity (A) of **4a** and **4j** at B3LYP/6–31 + G(d,p) level of theory

Entry	E_tot_^a^	H^a^	G^a^	S^b^	ɳ^c^	σ^d^	I^c^	A^c^
4a	− 949.3	− 949.3	− 949.3	139.9	2.560	0.391	6.994	1.874
4j	− 1004.6	− 1004.6	− 1004.7	150.1	2.453	0.408	6.916	2.010

^a^In Hartree/particle

^b^In cal/mol.K

^c^In ev

^d^In ev^−1^

**Fig. 15:**
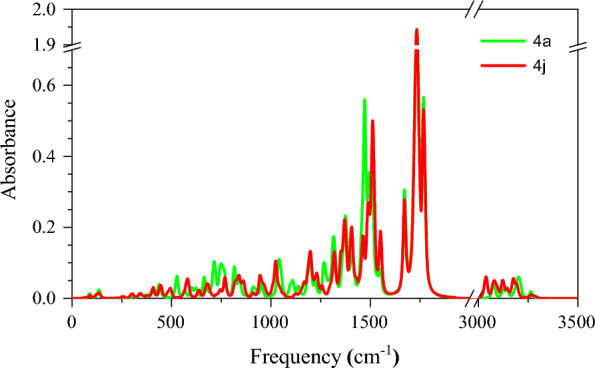
Calculated IR spectra for 4a and 4j at B3LYP/6–31 + G (d,p) level of theory

DFT calculations are a strong way to get precise and reliable IR spectra for diverse substances. The comparison of predicted and observed IR spectra serves to confirm the precision of DFT predictions, as well as the validity of molecule structure and properties. The IR spectra (Fig. 15) of C-C, C=C, C=N, C=O, and C-H aliphatic and aromatic were compared to experimental data. The results show that the predictions of DFT coincide with the actual data. As a result, **4a** and **4j** are computationally well-defined and characterized.

### ADME profile prediction

The oral bioavailability and drug-likeness of the synthesized compounds were predicted by using the SwissADME online program [41]. The results shown in Table 4 proved that the considered derivatives obey Rule of 5 Lipinski. Rotatable bands and TPSA were investigated to predict the oral bioavailability of the compounds which revealed that these parameters (TPSA ≤ 140 Å and nRB < 8) proved the appropriate values [42]. Overall, the studied ligands can be considered suitable drug-like candidates.

**Table 4:** ADME profile for synthesized compounds **(4a-4l)**

ID	M.W	Log p	nRB	HBA	HBD	TPSA	Lipinski	violation
4a	282.30	0.86	3	3	0	61.82	0	0
4b	296.32	1.11	3	3	0	61.82	0	0
4c	296.32	1.11	3	3	0	61.82	0	0
4d	361.19	1.5	3	3	0	61.82	0	0
4e	296.32	1.11	3	3	0	61.82	0	0
4f	310.35	1.35	3	3	0	61.82	0	0
4g	375.22	1.74	3	3	0	61.82	0	0
4h	283.29	1.02	3	4	0	74.71	0	0
4i	297.31	1.28	3	4	0	74.71	0	0
4j	297.31	1.28	3	4	0	74.71	0	0
4k	362.18	1.67	3	4	0	74.71	0	0
4l	269.26	1.03	3	4	0	74.71	0	0

The pharmacokinetic properties of the synthesized compounds and Erlotinib are presented in Table 5. The results predicted that the compounds **4a** and **4h-4l** had no neurotoxicity. Overexpression of P-gp in cancer cells is responsible for multidrug resistance (MDR), and to pump the anticancer drugs outside the cell. The data showed that all compounds were not P‐glycoprotein substrates. All compounds were CYP1A2 inhibitors whereas had no inhibitory effect on CYP2D6. The CYP2C19, CYP2C9, and CYP3A4 inhibitory effects for the studied compounds were different. The compounds **4b-4g** had no inhibitory effect on CYP2C19 and the compounds **4d, 4f** and **4g** were CYP2C9 inhibitors. The predictions indicated that the compounds **4b, 4c, 4e, 4f,** and **4g** had CYP3A4 inhibitory.

**Table 5:** Pharmacokinetic properties for synthesized compounds **(4a-4l)**

Entry	BBB permeation	P-gp substrate	CYP1A2 inhibitor	CYP2C19 inhibitor	CYP2C9 inhibitor	CYP2D6 inhibitor	CYP3A4 inhibitor
4a	No	No	Yes	Yes	No	No	No
4b	Yes	No	Yes	No	No	No	Yes
4c	Yes	No	Yes	No	No	No	Yes
4d	Yes	No	Yes	No	Yes	No	No
4e	Yes	No	Yes	No	No	No	Yes
4f	Yes	No	Yes	No	Yes	No	Yes
4g	Yes	No	Yes	No	Yes	No	Yes
4h	No	No	Yes	Yes	No	No	No
4i	No	No	Yes	Yes	No	No	No
4j	No	No	Yes	Yes	No	No	No
4k	No	No	Yes	Yes	No	No	No
4l	No	No	Yes	Yes	No	No	No
Erlotinib	Yes	No	Yes	Yes	Yes	Yes	Yes

## Conclusion

A series of uracil-azole derivatives were designed, synthesized, and fully characterized with ^1^HNMR, ^13^CNMR, and Mass spectroscopy. The antiproliferative activities of these 12 newly synthesized uracil-azole derivatives were evaluated against two human tumor cell lines including MCF-7 (Breast Cancer), and HEPG-2 (Hepatocellular Cancer). Compound **4j** was the most potent compound with IC_50_ values of 16.18 ± 1.02 and 7.56 ± 5.28 in MCF-7, and HEPG-2 cell lines compared to Cisplatin with IC_50_ values of 20.70 ± 0.83 and 15.91 ± 1.83. Structure–activity relationship reveals that triazole and electro donating group at benzyl moiety increased antiproliferative activity. Molecular docking, the binding interaction and energy of the molecule in an active site of EGFR target was confirmed the biological activity. Investigation of reactivity descriptors by using DFT calculations on **4a** and **4j**, shows that the **4j** is softer and more reactive compound. Furthermore, the addition of the methyl group in **4j** increases entropy and, as a result, molecule softness. In addition, the HOMO and LUMO of these molecules (**4a** and **4j**) are strongly influenced by the position of the N atom in the imidazole or triazole rings. The frequency calculation confirms that molecules are stationary, and the resulting IR spectra agree with experimental data. Based on these results, uracil-azole derivatives could be considered as a promising candidate for anticancer drug discovery.

### Supplementary Information

**Additional file 1.** This file contains the analytical data of the synthesized compounds such as ^1^HNMR and ^13^C-NMR, Mass and FT-IR spectra.

## Data Availability

The data sets used and analyzed during the current study are available from the corresponding author upon reasonable request. We have presented all data in the form of Tables and Figures. The PDB code (1M17) was retrieved from the protein data bank (www.rcsb.org). https://www.rcsb.org/structure/1M17.

## Abbreviations

SAR: Structure-activity relationship
ADME: Adsorption, distribution, metabolism, and excretion
DFT: Density functional theory
MTT: Standard 3-(4,5-dimethylthiazol-yl)-2,5-diphenyl-tetrazolium bromide
EGFR: Epidermal growth factor receptor
HOMO: Highest occupied molecular orbital
LUMO: Least unoccupied molecular orbital
MD: Molecular dynamic
TPSA: Topological polar surface area
