# Supplementary material for: Synthesis, design, biological evaluation, and computational analysis of some novel uracil-azole derivatives as cytotoxic agents
Source: BMC Chem. 2024 Jan 3;18(1):3. doi: 10.1186/s13065-023-01106-x (PMC10765869; doi:10.1186/s13065-023-01106-x)
Supplement: Supplementary file 1 — Additional file 1. This file contains the analytical data of the synthesized compounds such as 1HNMR and 13C-NMR, Mass and FT-IR spectra. [file 13065_2023_1106_MOESM1_ESM.docx]

**Synthesis, Design, Biological Evaluation, and Computational Analysis of Some Novel Uracil-Azole Derivatives as Cytotoxic Agents**

Leila Emami^1a^, Fateme Zare^2a^, Soghra Khabnadideh^1^, Zahra Rezaei^1^, Zahra Sabahi^2^, Saman Zare Gheshlaghi^3^, Marzieh Behrouz^4^, Mina Emami^1^, Zahra Ghobadi^2^, Sedighe Madadelahi Ardekani^1^, Fatemeh Barzegar^1^, Ali Ebrahimi^3^, Razieh Sabet ^[[1]](#footnote-1)^* ^2^

*^1^Pharmaceutical Sciences Research Center, Shiraz University of Medical Sciences, Shiraz, Iran*

*^2^Department of Medicinal Chemistry, Faculty of Pharmacy, Shiraz University of Medical Sciences, Shiraz, I.R. Iran.*

*^3^ Department of Chemistry, Computational Quantum Chemistry Laboratory, University of Sistan and Baluchestan, Zahedan, Iran*

*^4^ Department of Chemistry, Shiraz University of Technology, Shiraz, Iran*


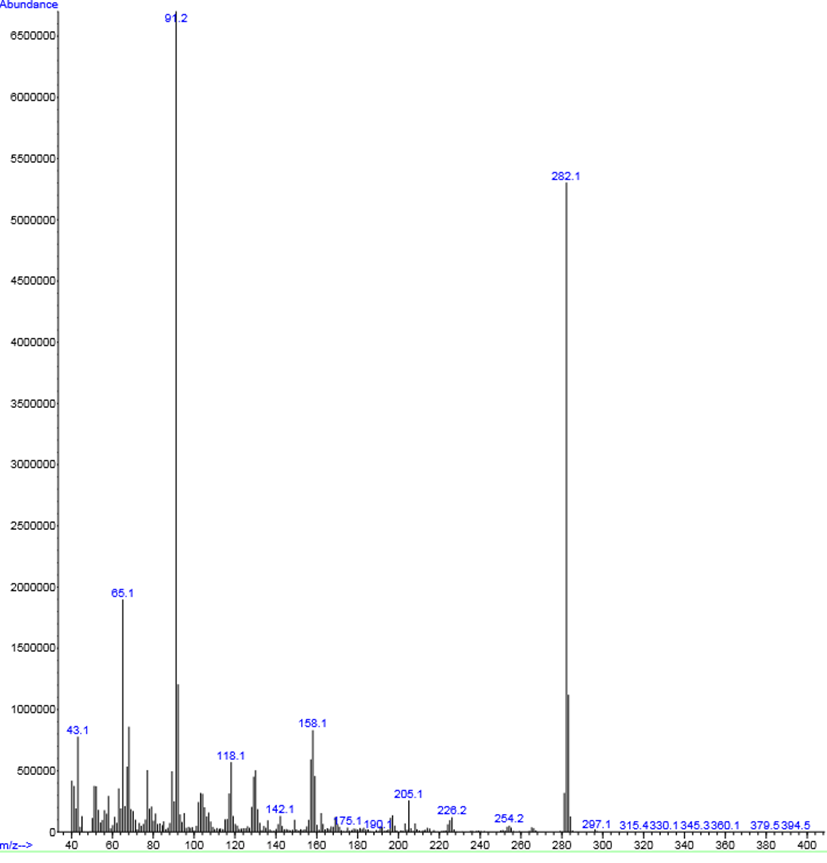


S_1_: Mass spectrum of 4a


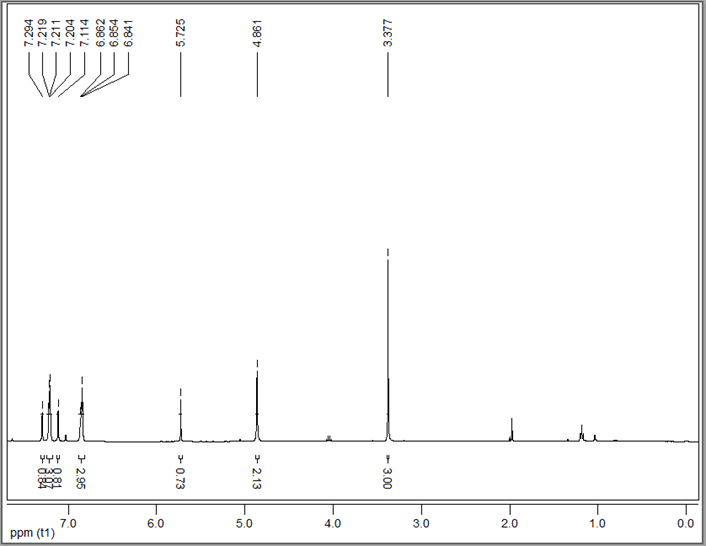


S_2_: ^1^HNMR spectrum of 4a


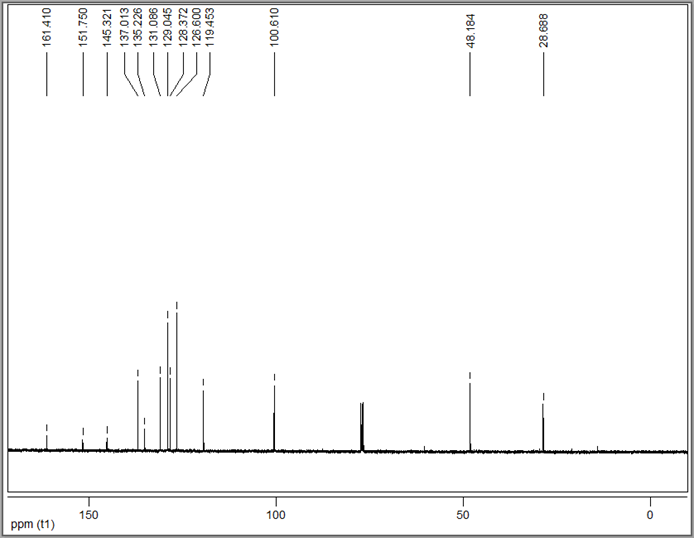


S_3_: ^13^CNMR spectrum of 4a


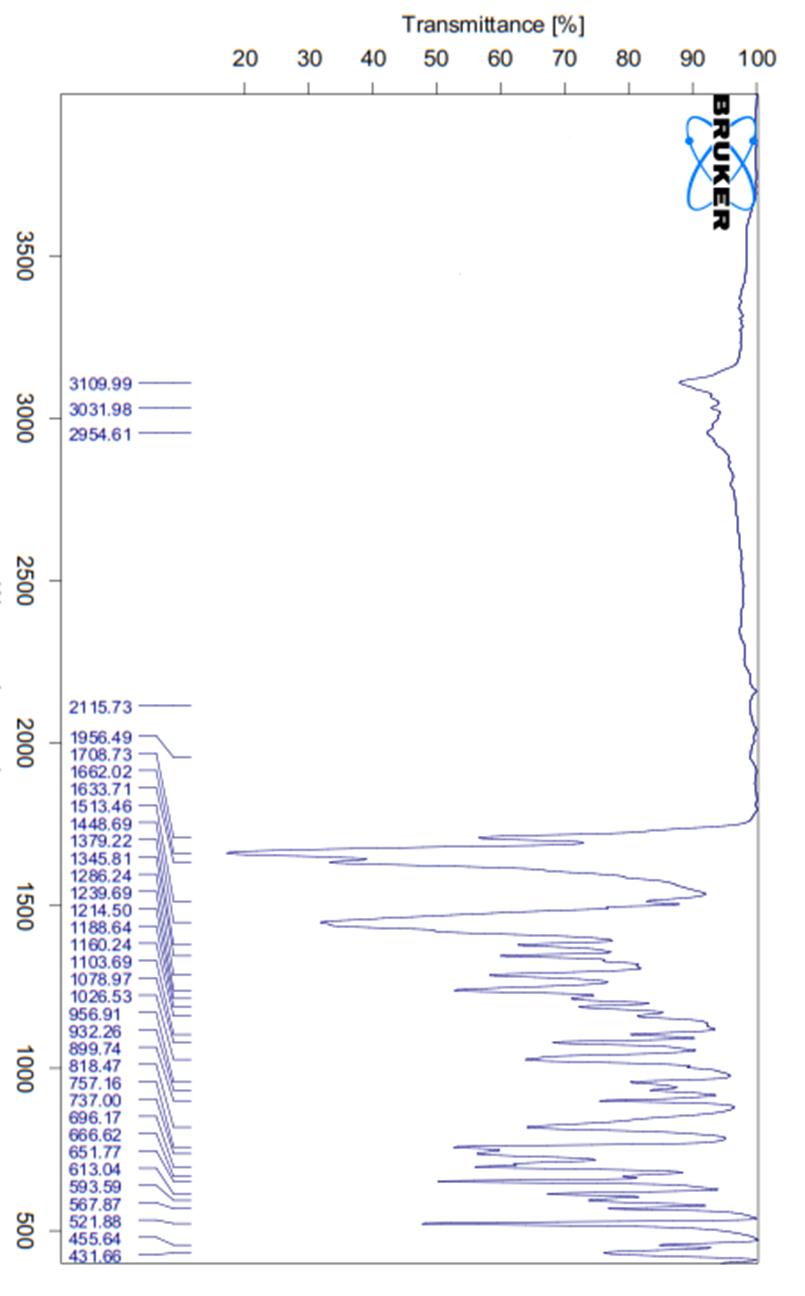


S_4_: IR spectrum of 4a


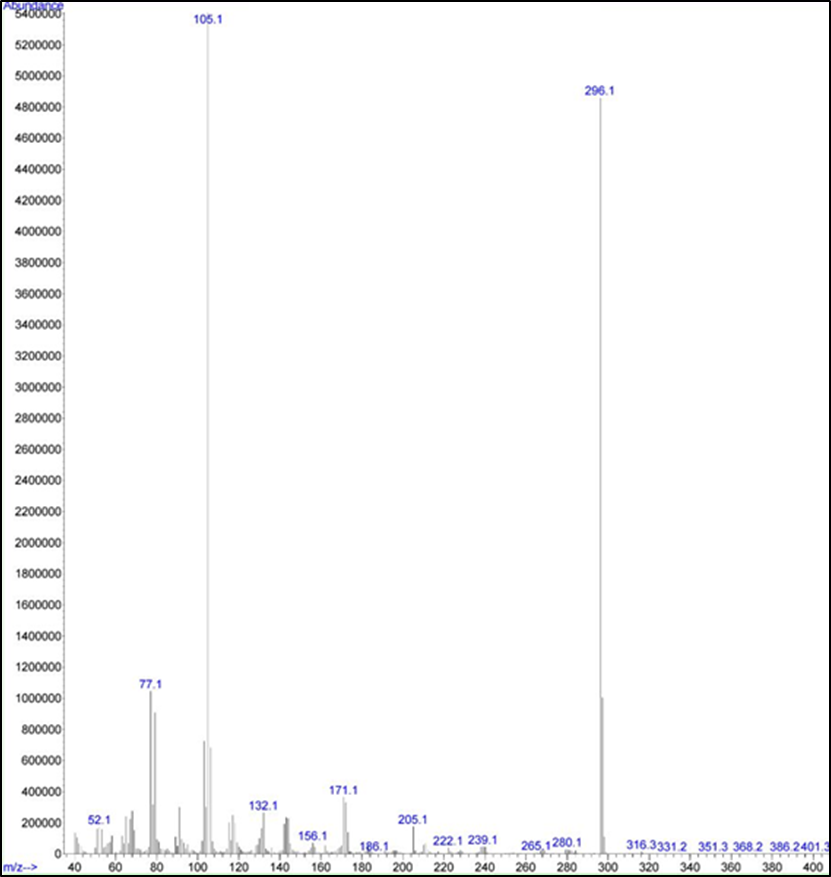


S_5_: Mass spectrum of 4b


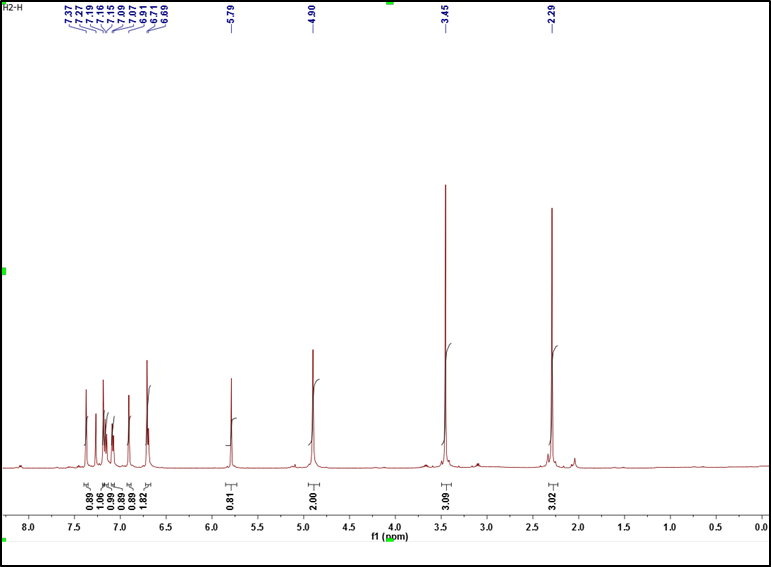


S_6_: ^1^HNMR spectrum of 4b


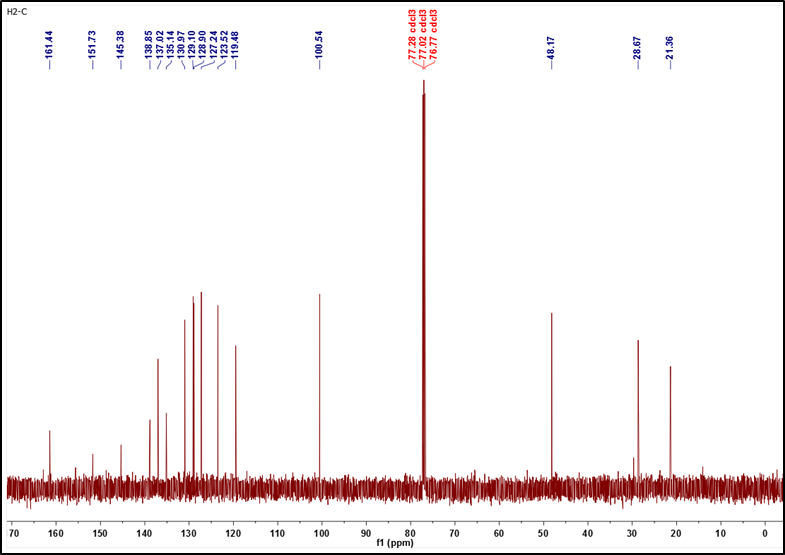


S_7_: ^13^CNMR spectrum of 4b


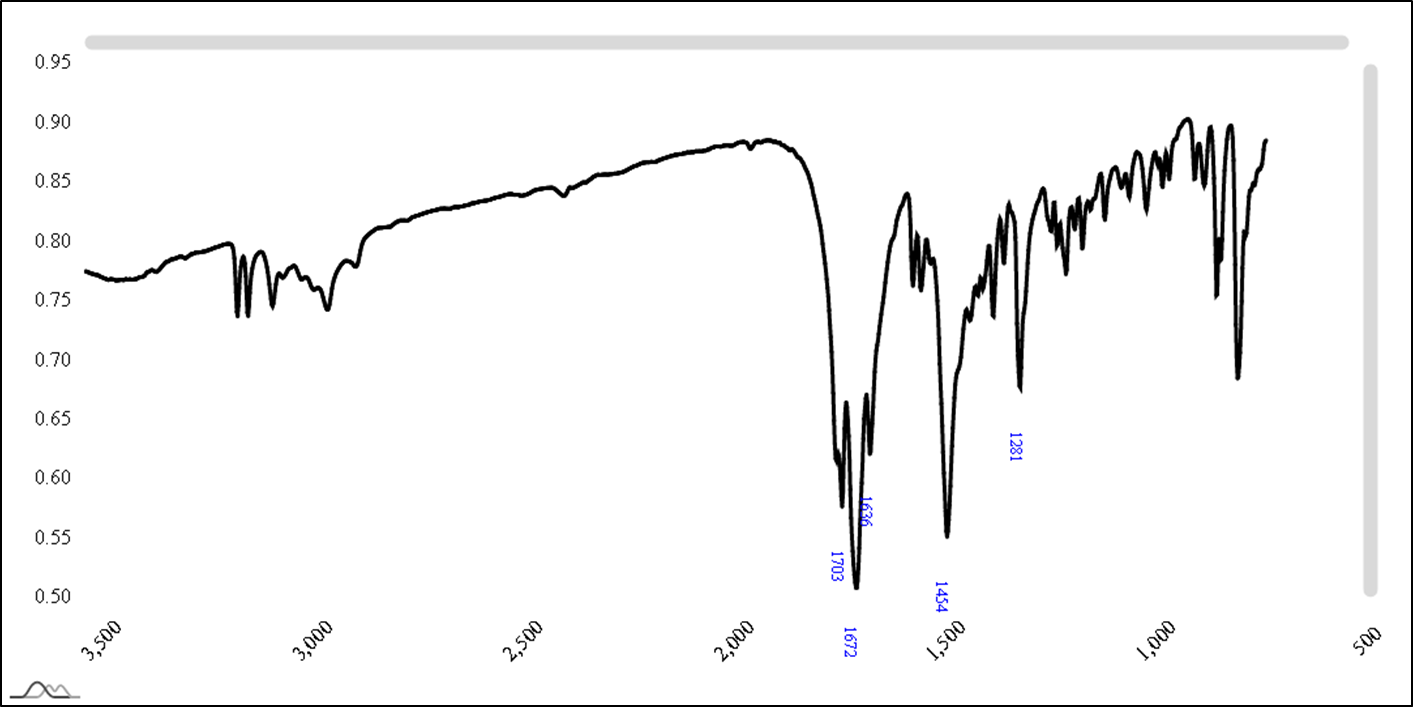


S_8_: IR spectrum of 4b


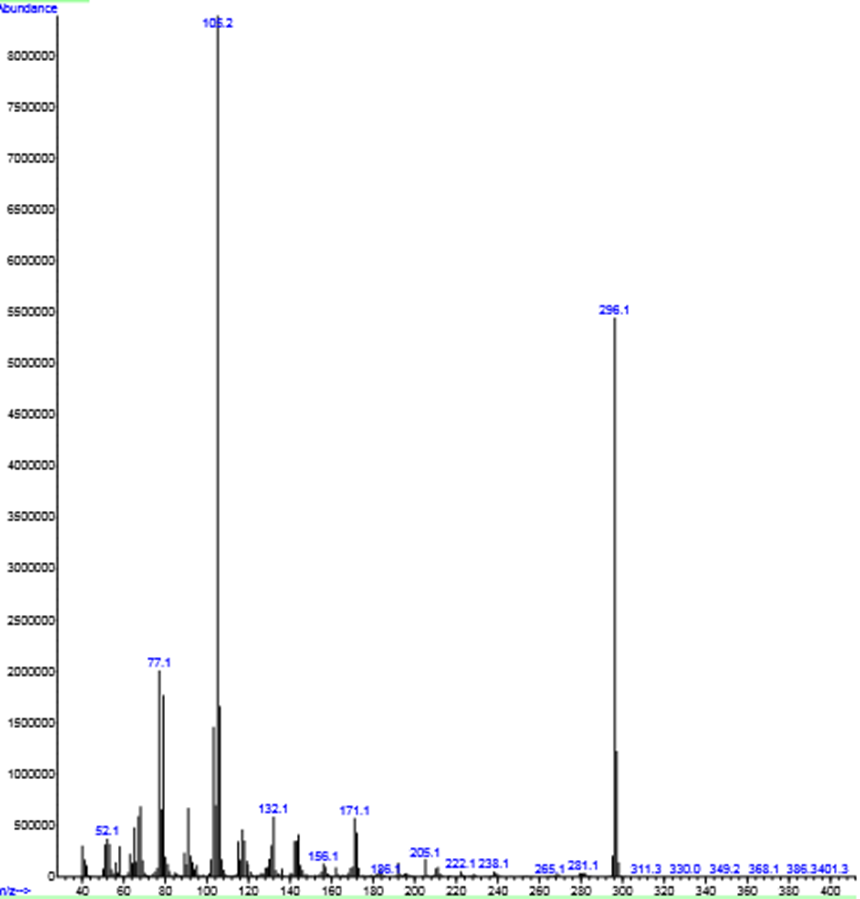


S_9_: Mass spectrum of 4c


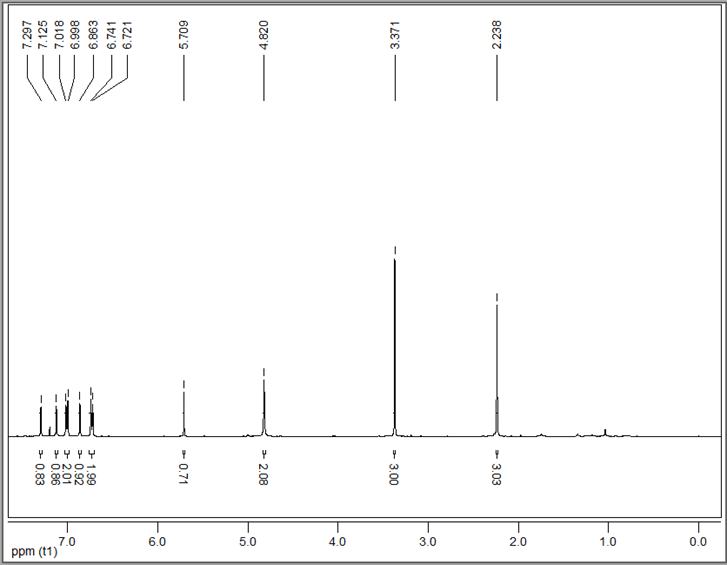


S_10_: ^1^HNMR spectrum of 4c


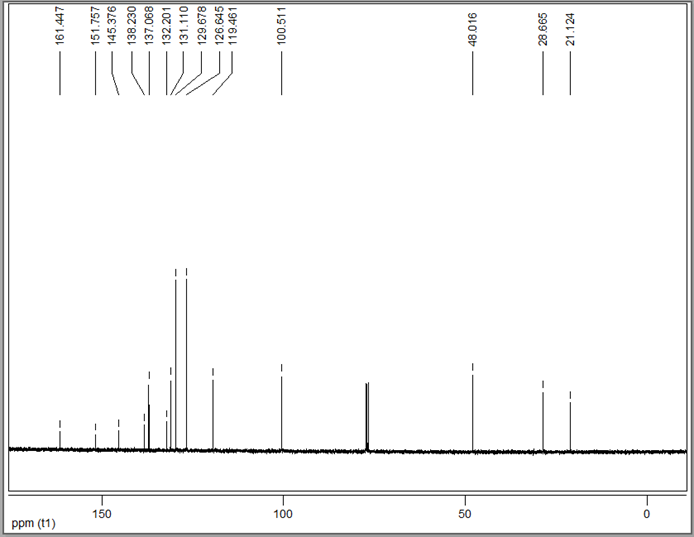


S_11_: ^13^CNMR spectrum of 4c


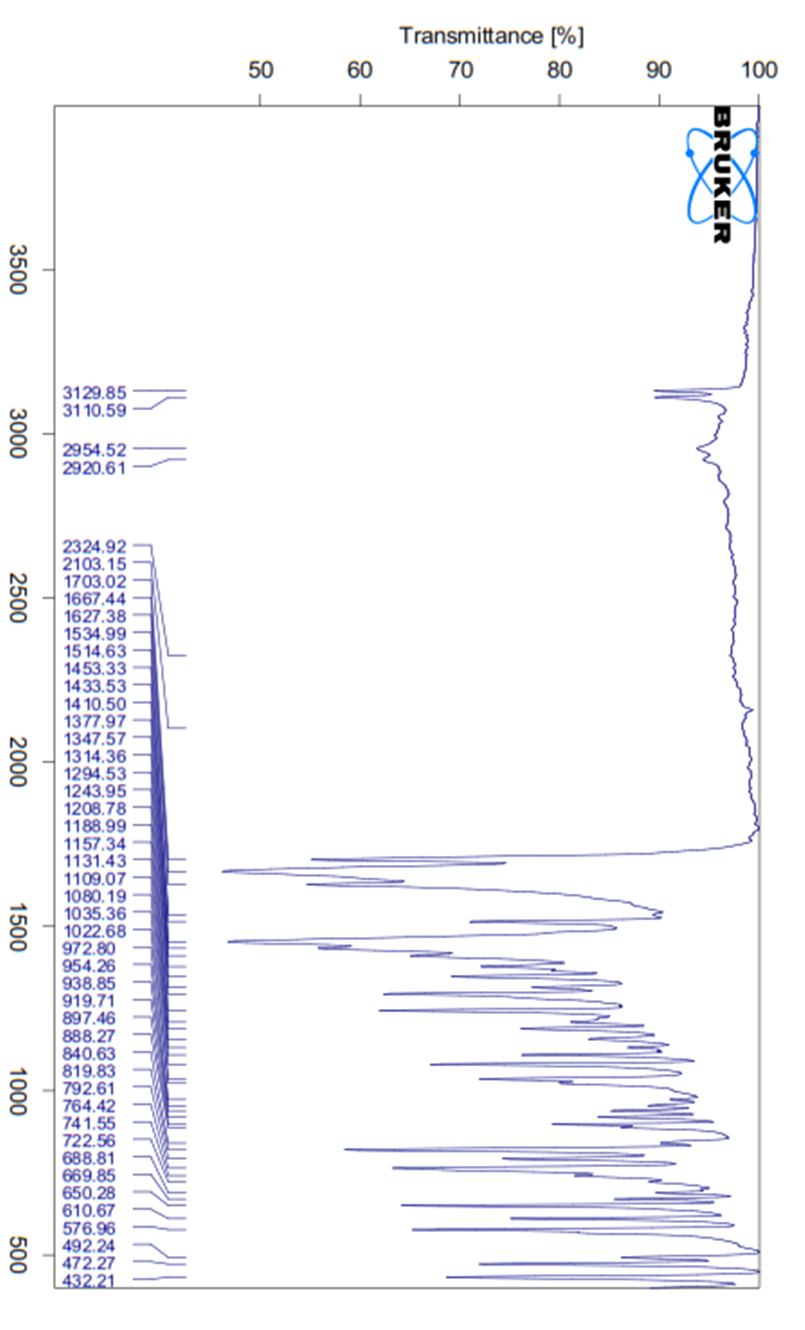


S_12_: IR spectrum of 4c


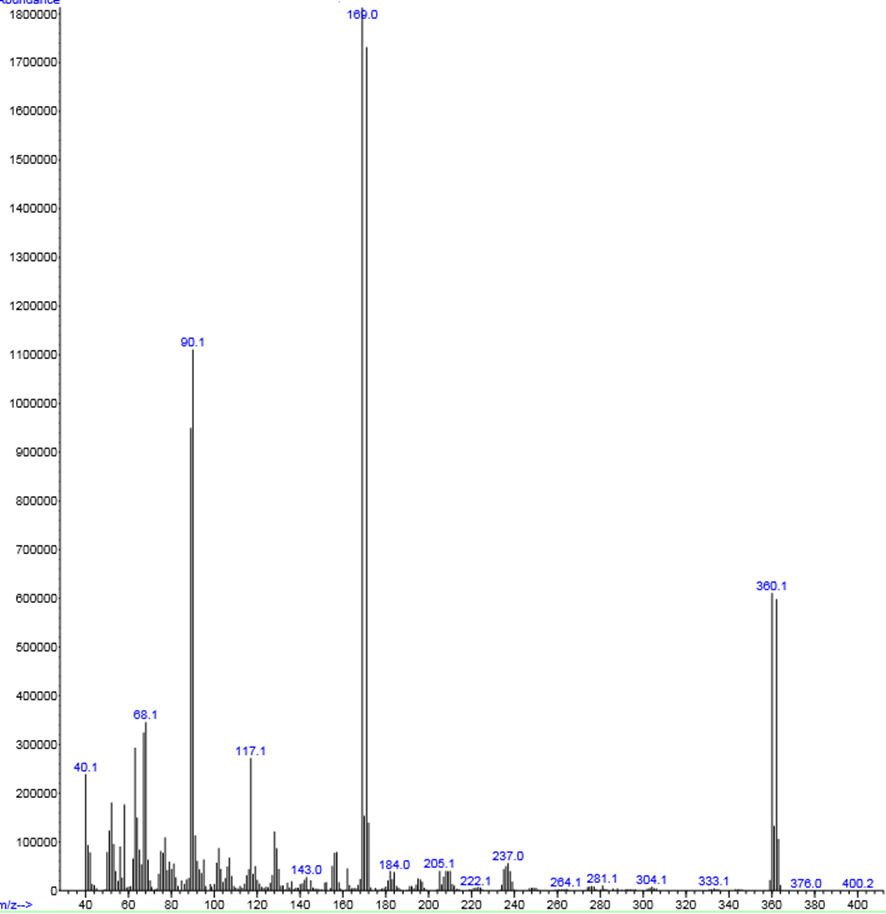


S_13_: Mass spectrum of 4d


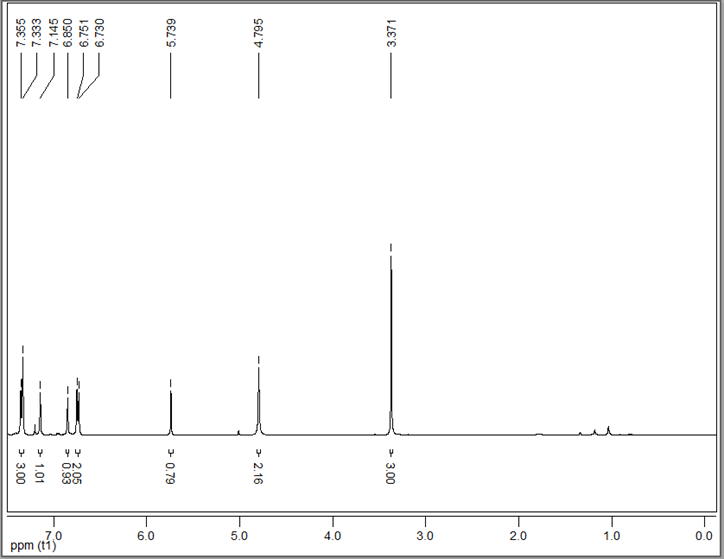


S_14_: ^1^HNMR spectrum of 4d


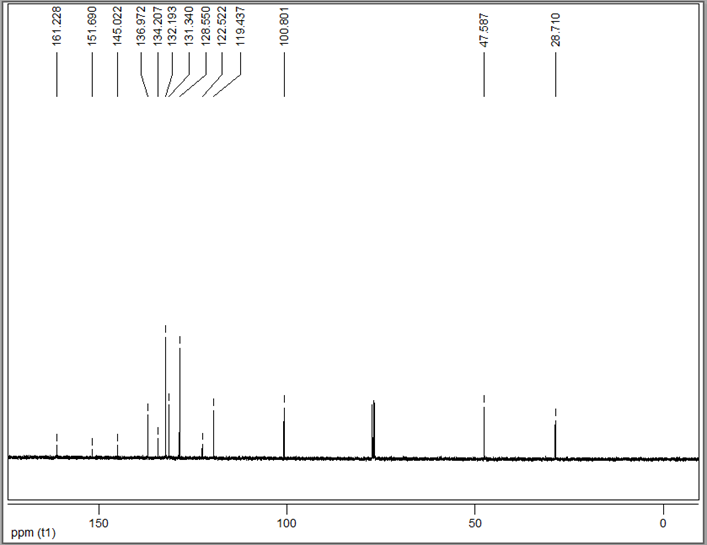


S_15_: ^13^CNMR spectrum of 4d


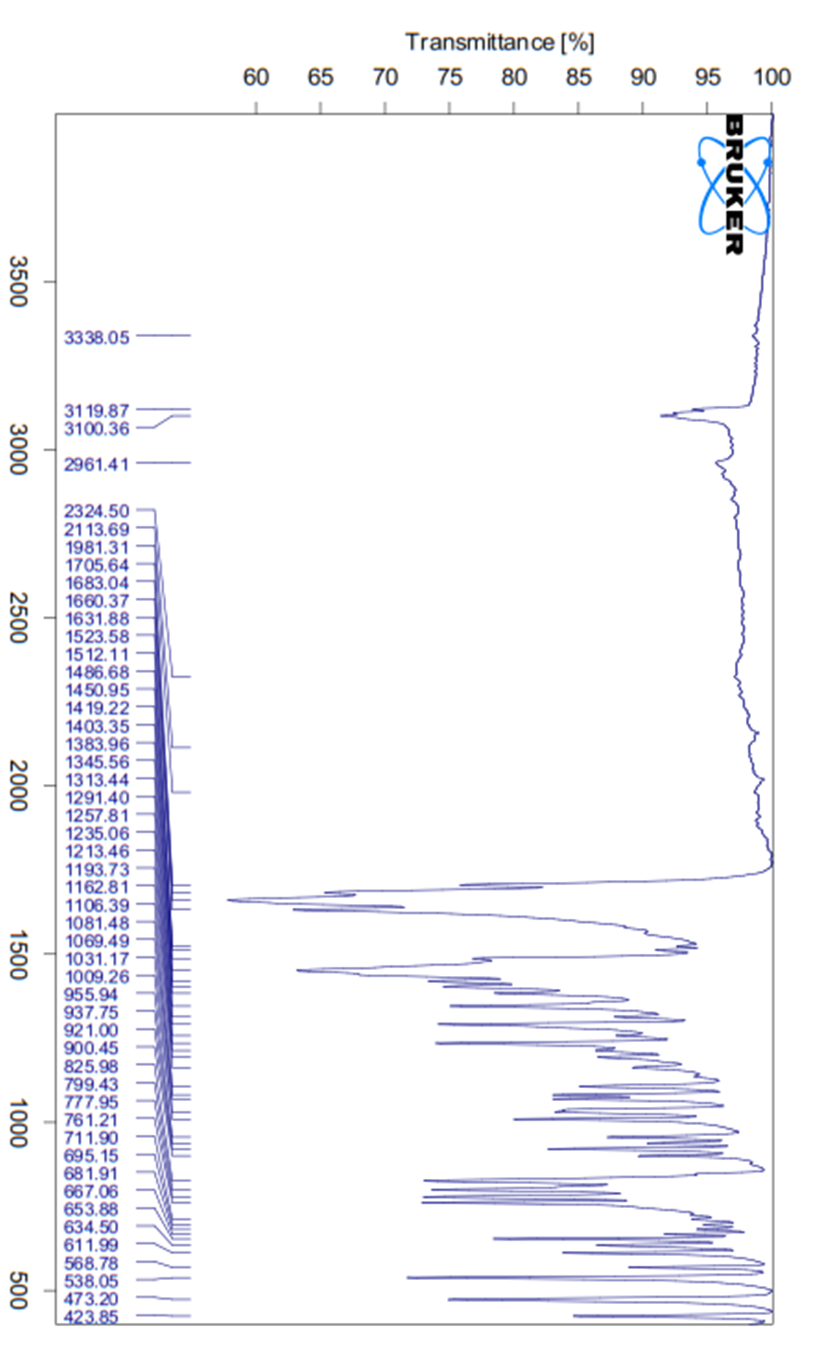


S_16_: IR spectrum of 4d


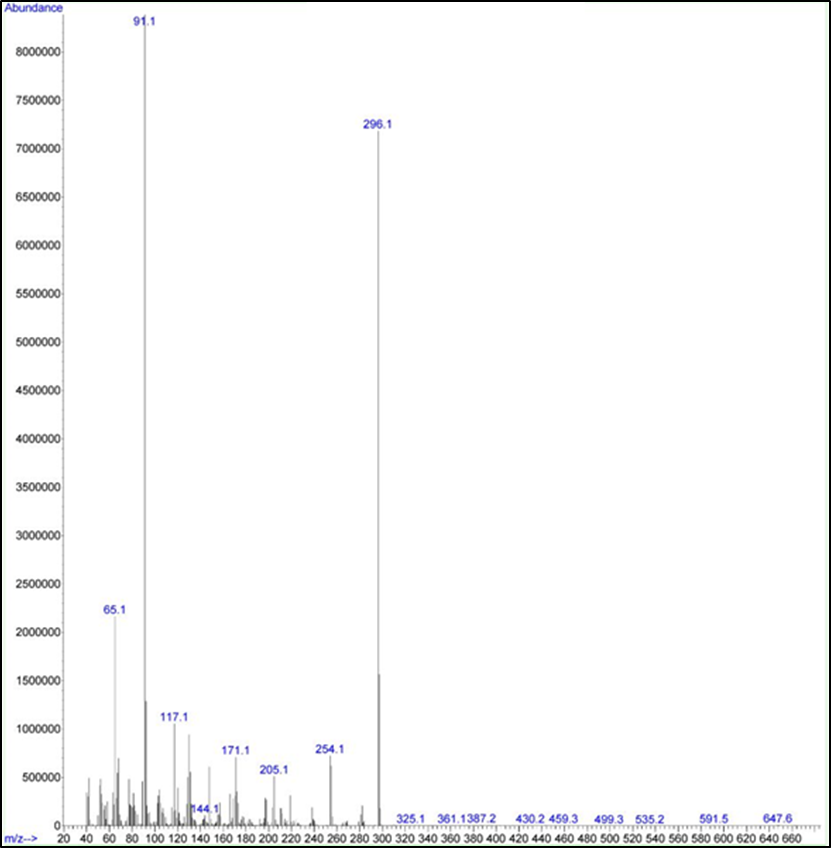


S_17_: Mass spectrum of 4e


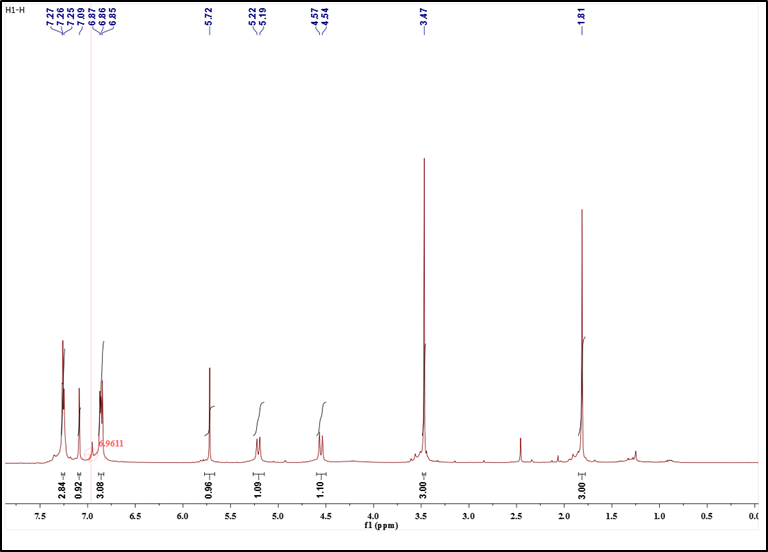


S_18_: ^1^HNMR spectrum of 4e


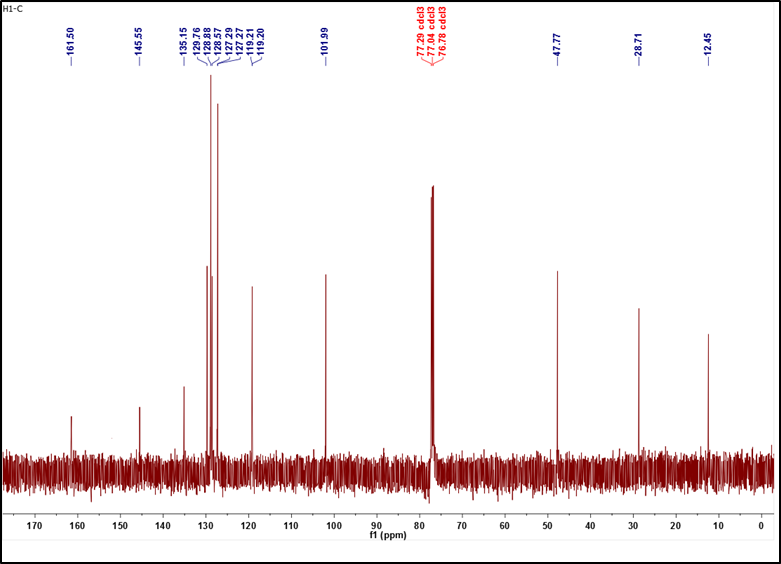


S_19_: ^13^CNMR spectrum of 4e


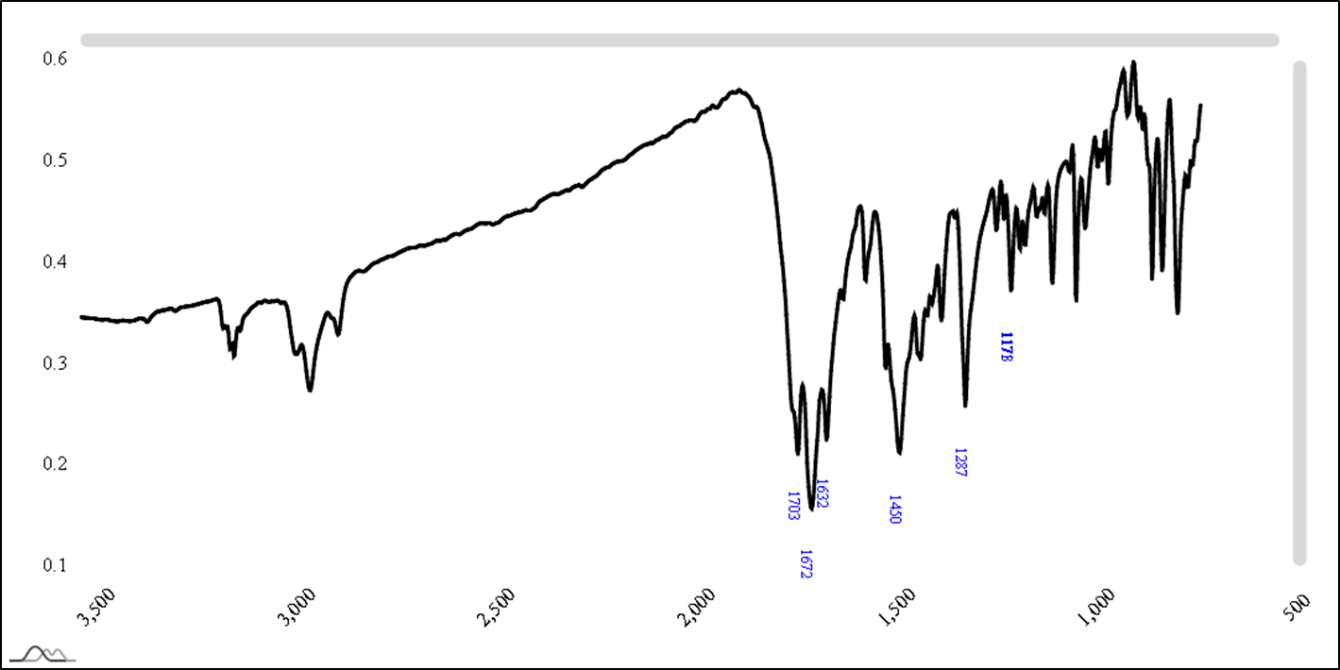


S_20_: IR spectrum of 4e


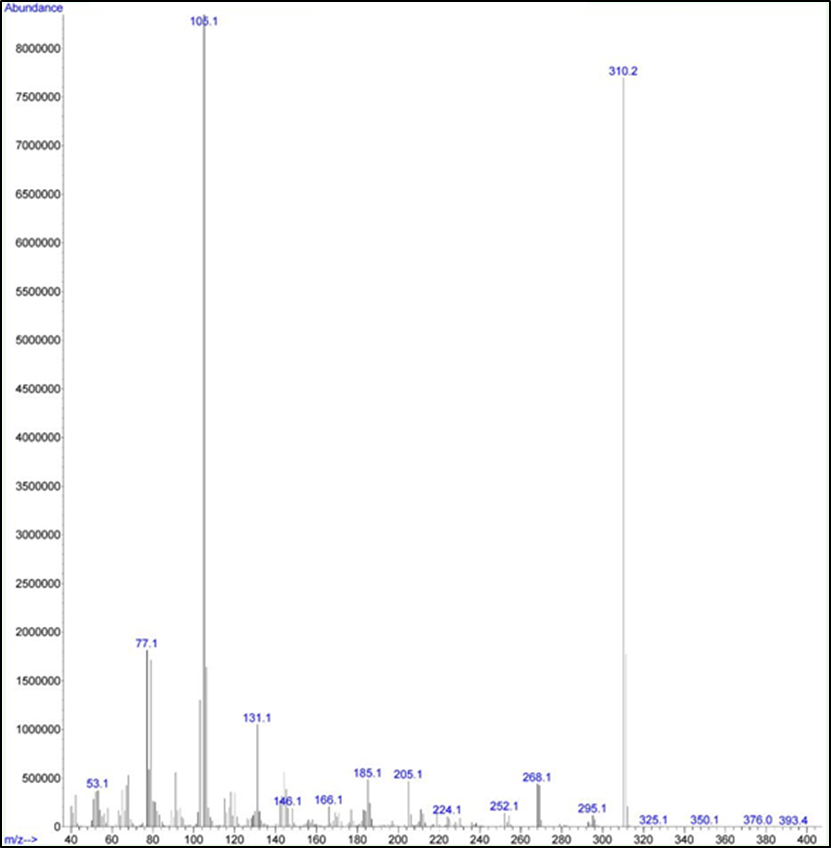


S_21_: Mass spectrum of 4f


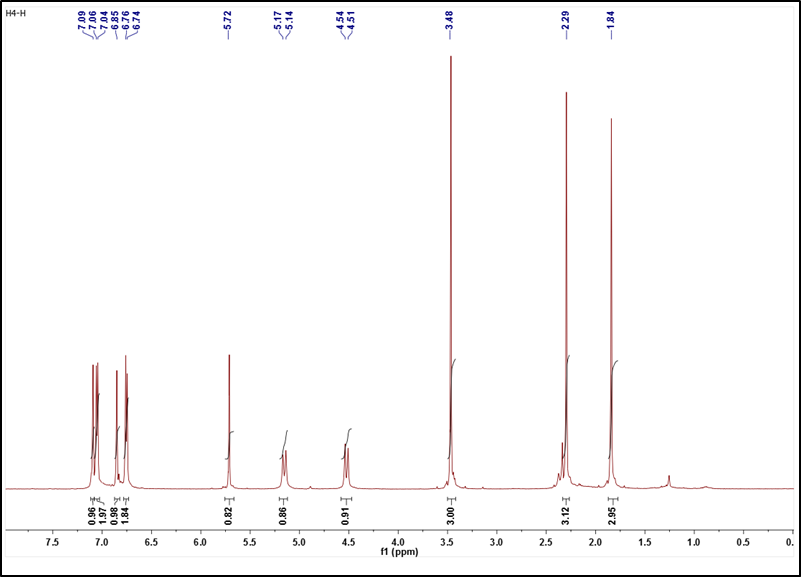


S_22_: ^1^HNMR spectrum of 4f


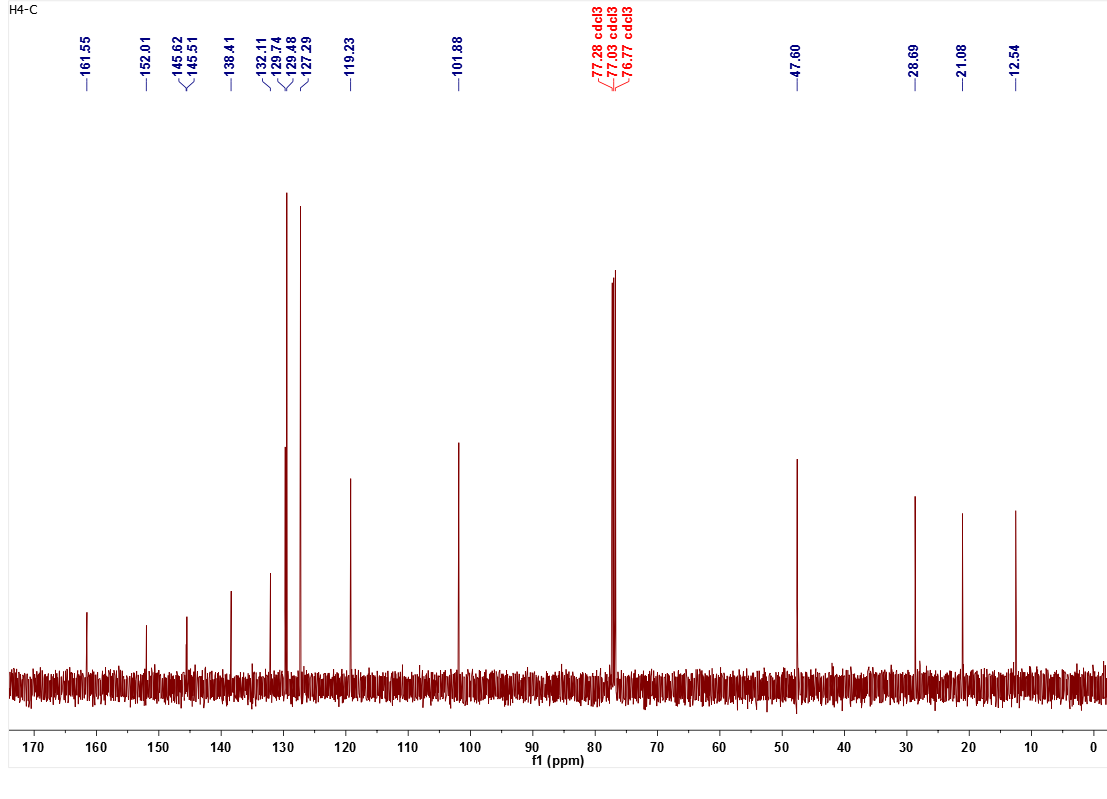


S_23_: ^13^CNMR spectrum of 4f


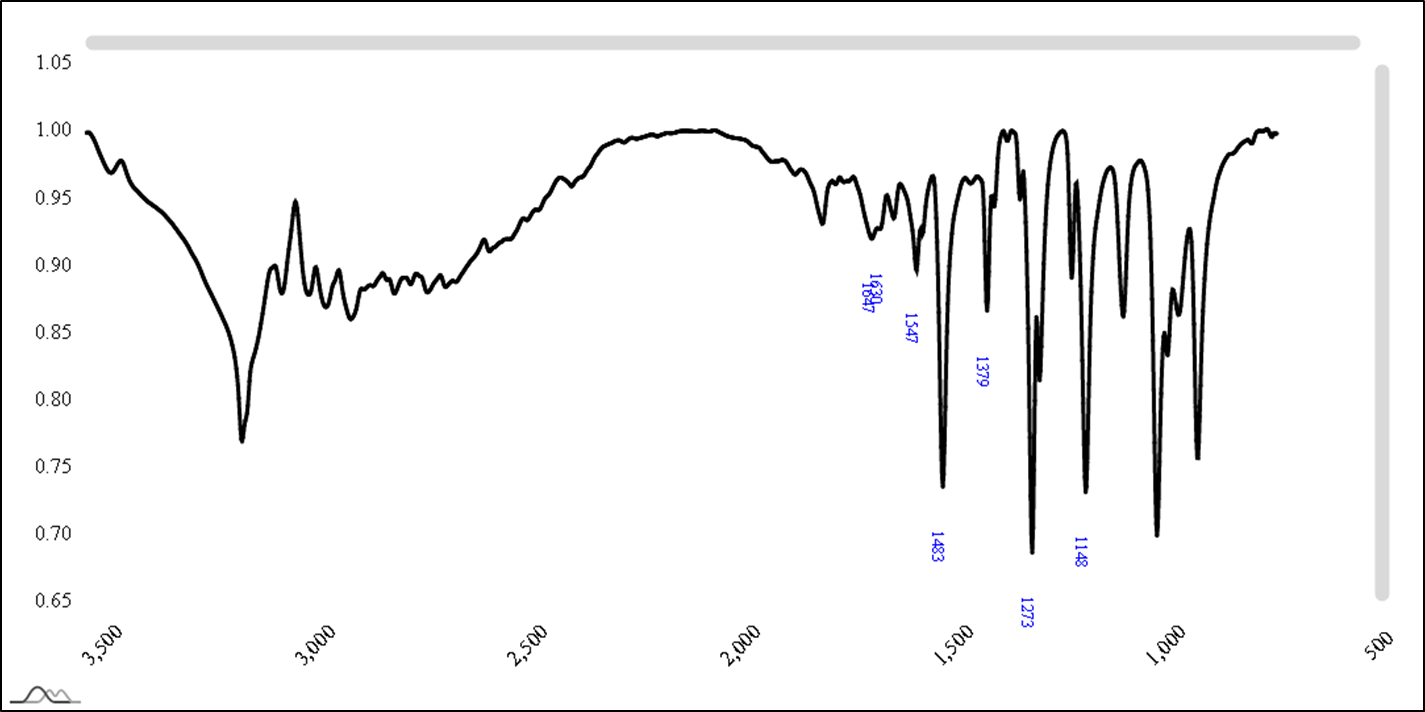


S_24_: IR spectrum of 4f


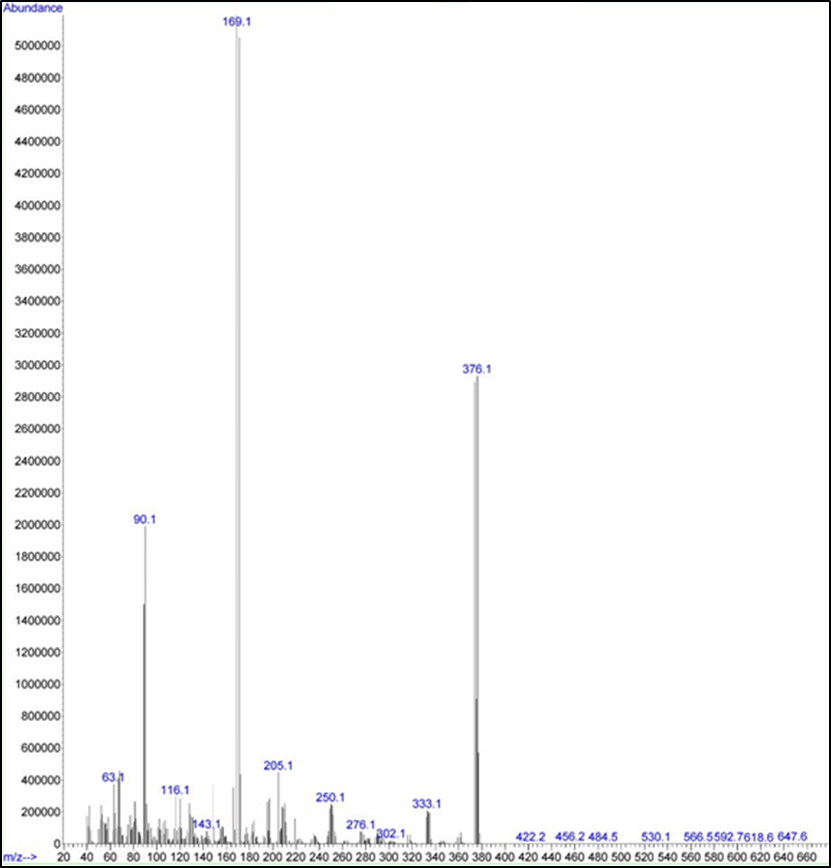


S_25_: Mass spectrum of 4g


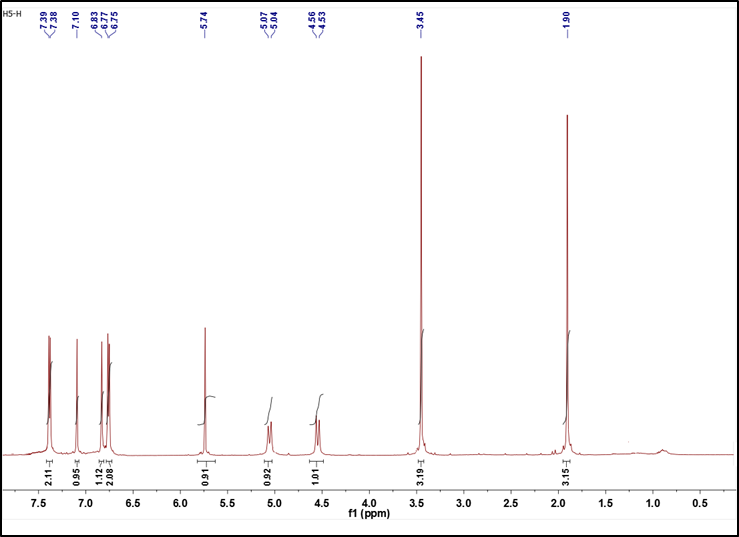


S_26_: ^1^HNMR spectrum of 4g


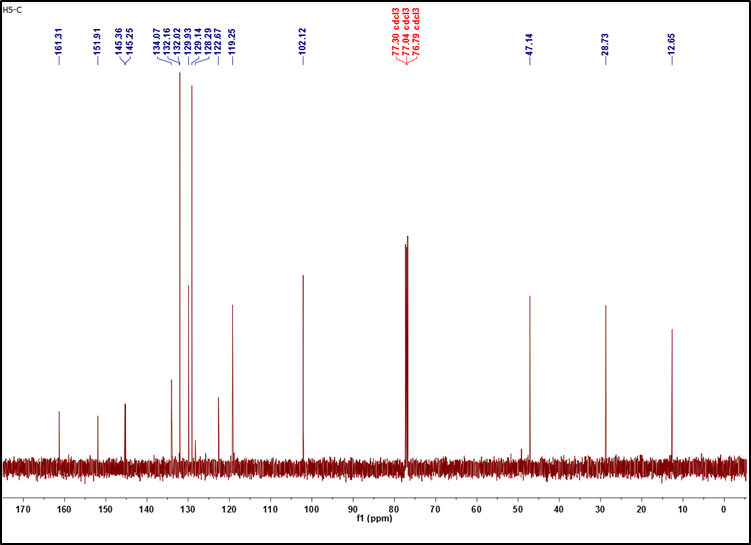


S_27_: ^13^CNMR spectrum of 4g


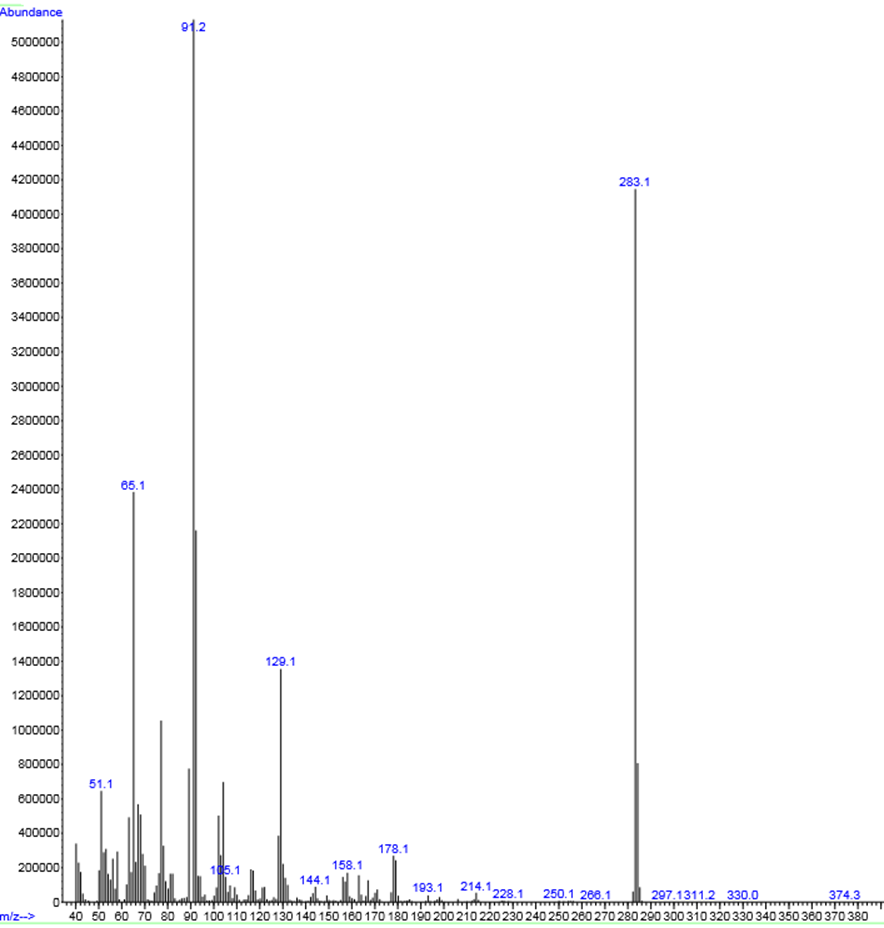


S_28_: Mass spectrum of 4h


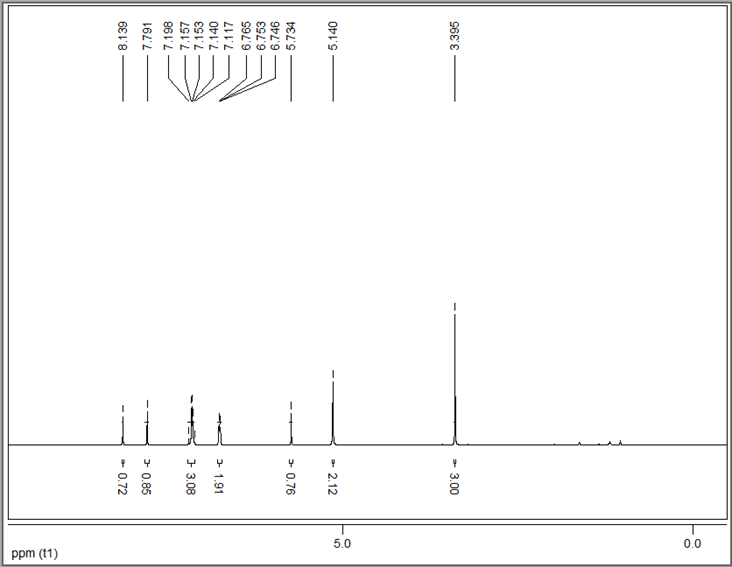


S_29_: ^1^HNMR spectrum of 4h


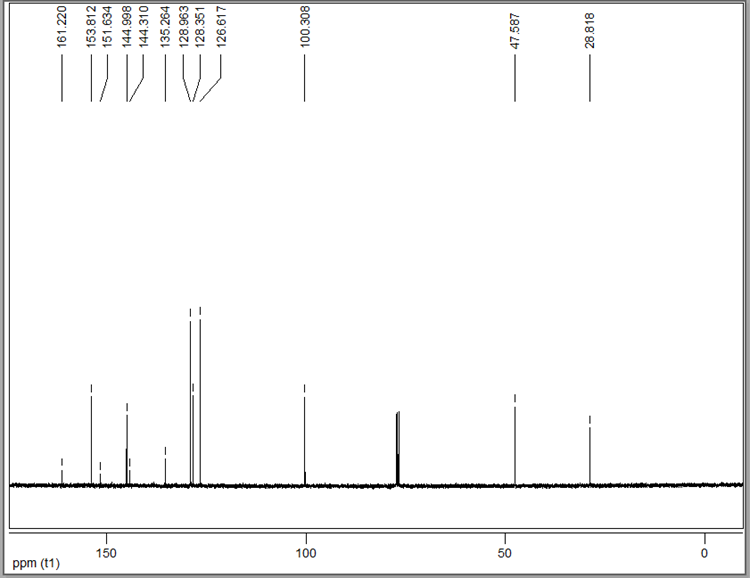


S_30_: ^13^CNMR spectrum of 4h


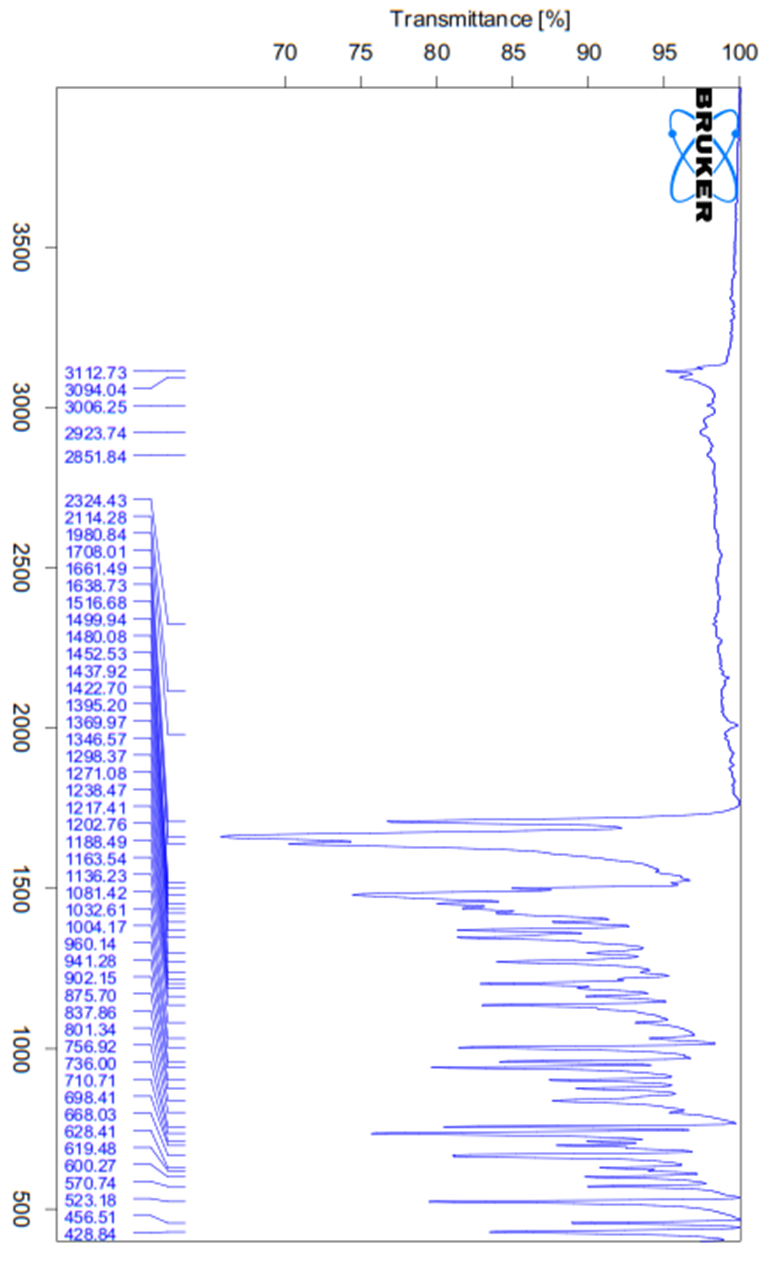


S_31_: IR spectrum of 4h


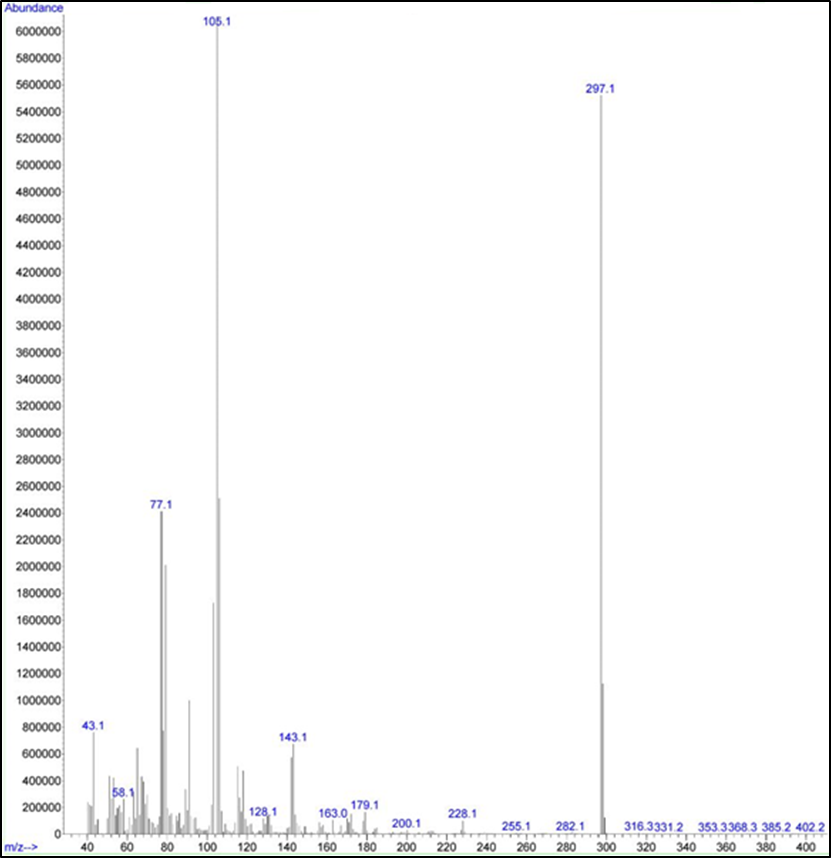


S_32_: Mass spectrum of 4i


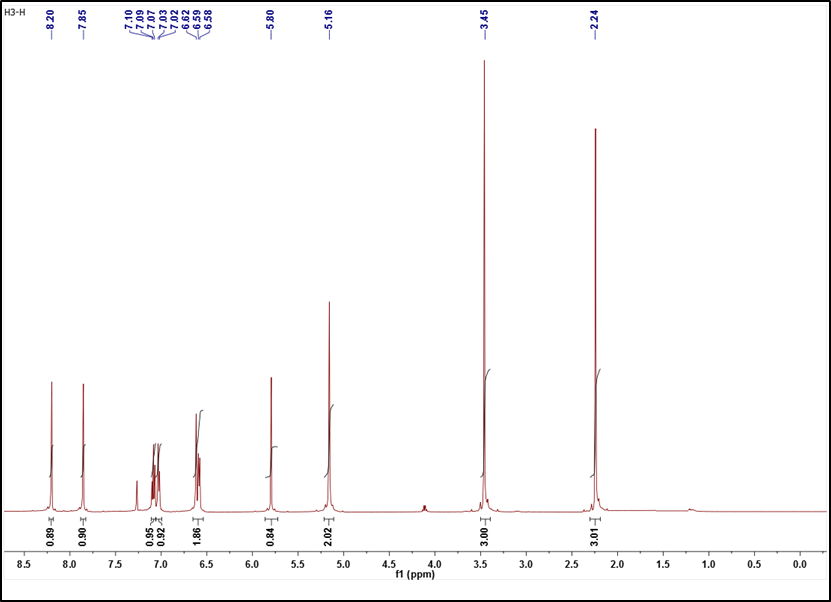


S_33_: ^1^HNMR spectrum of 4i


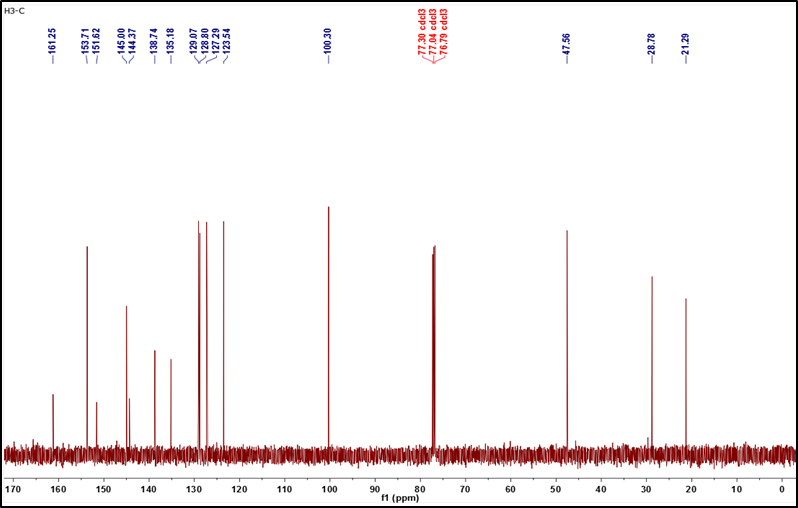


S_34_: ^13^CNMR spectrum of 4i


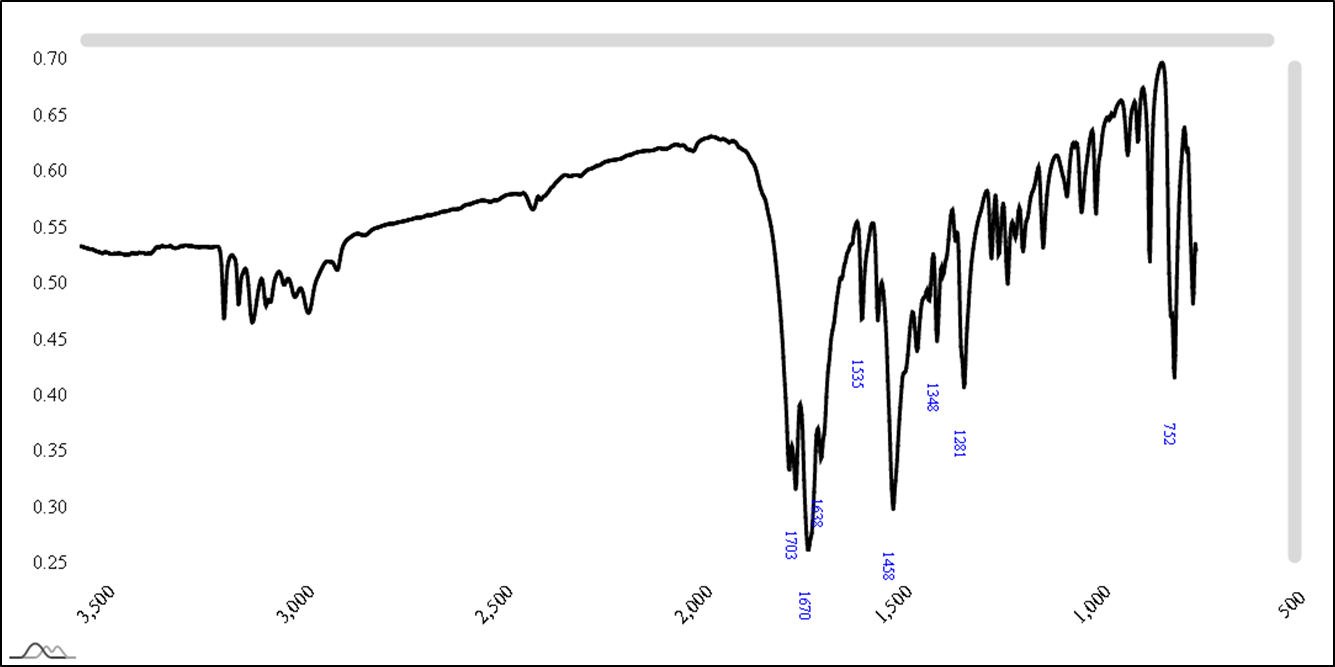


S_35_: IR spectrum of 4i


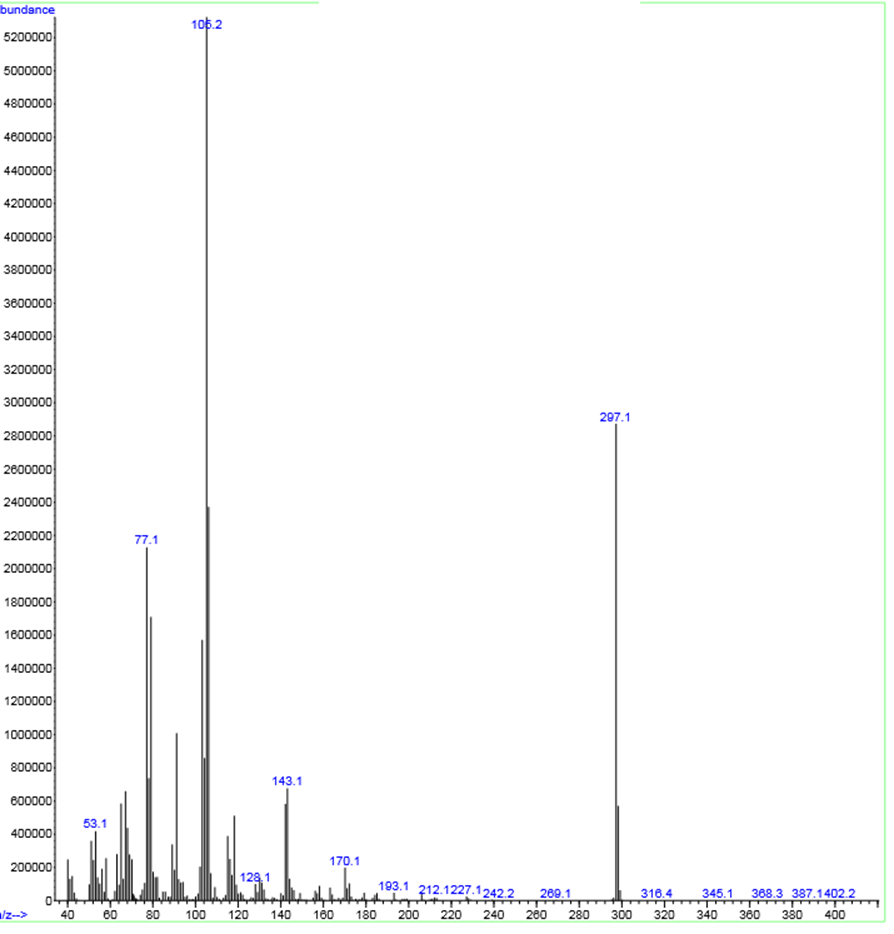


S_36_: Mass spectrum of 4j


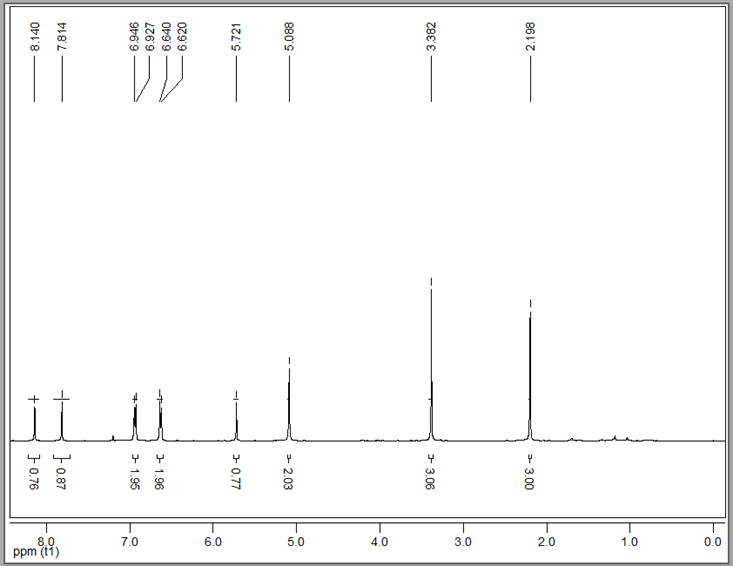


S_37_: ^1^HNMR spectrum of 4j


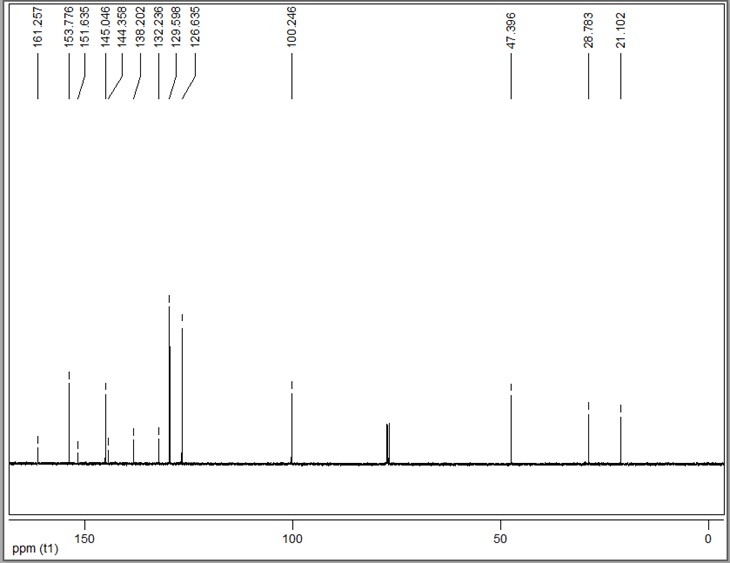


S_38_: ^13^CNMR spectrum of 4j


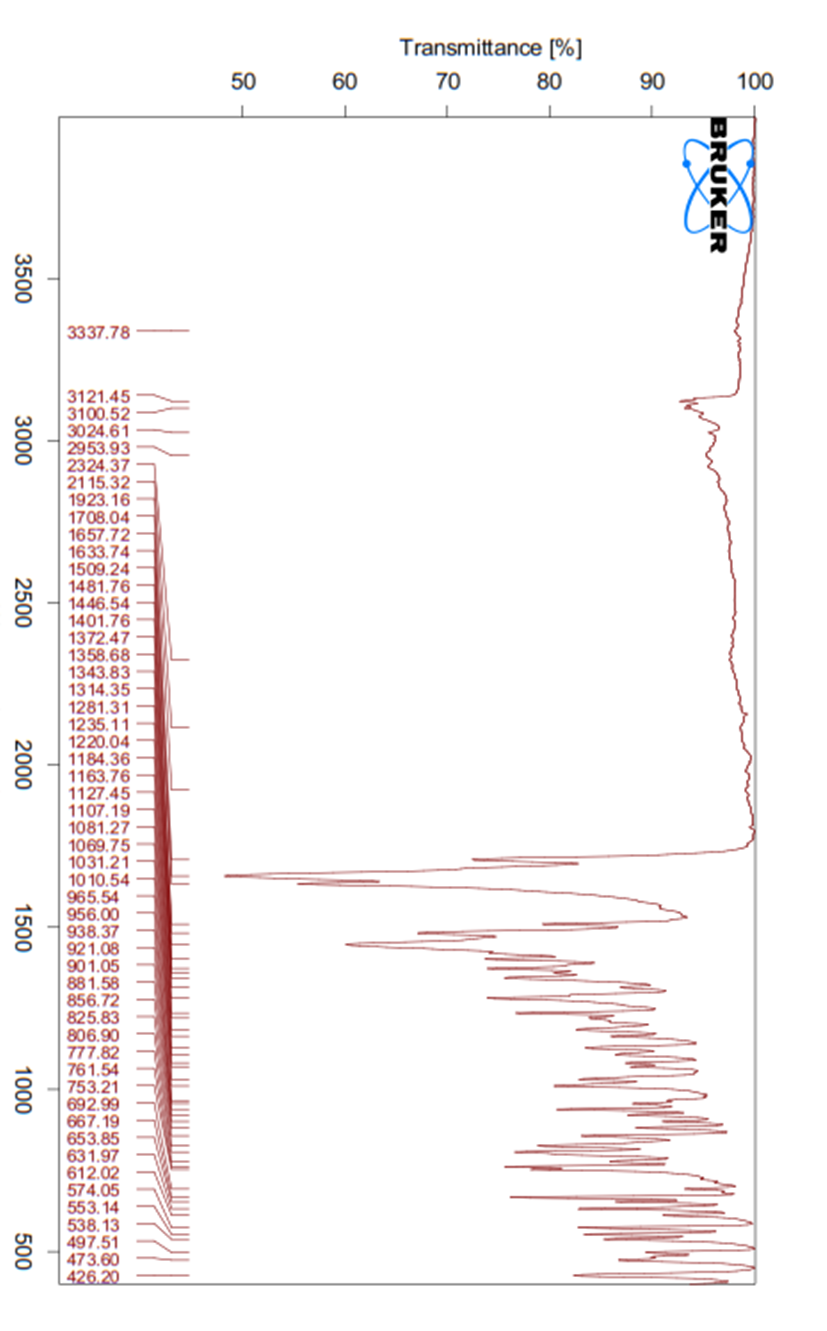


S_39_: IR spectrum of 4j


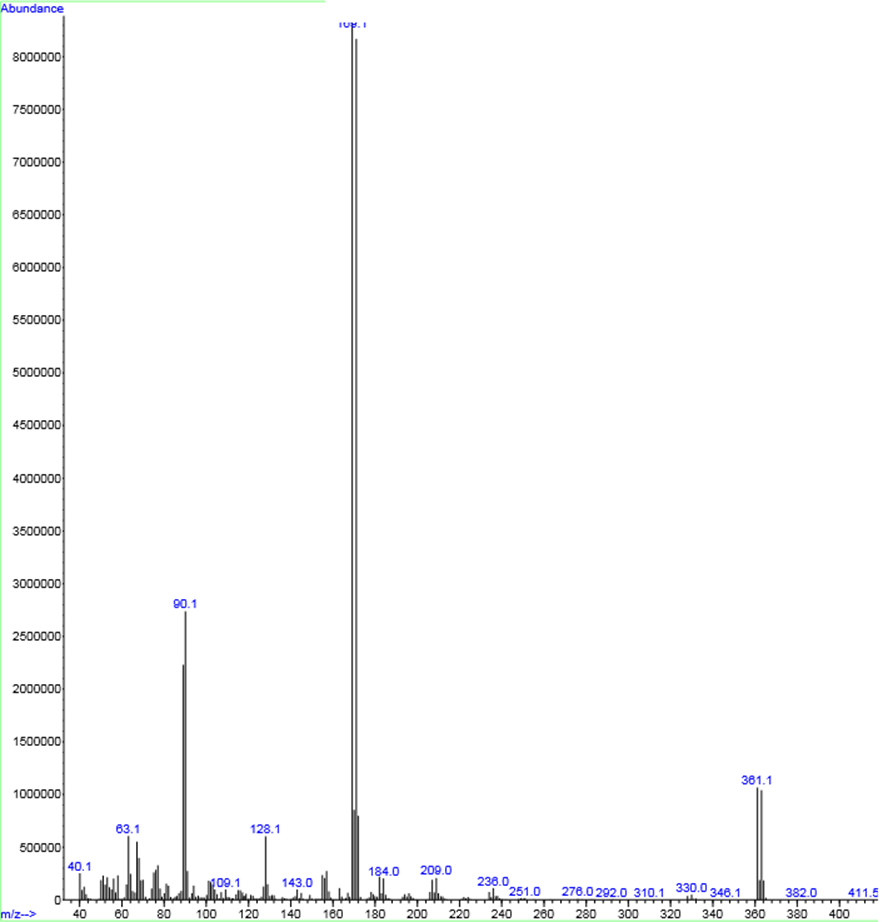

S_40_: Mass spectrum of 4k


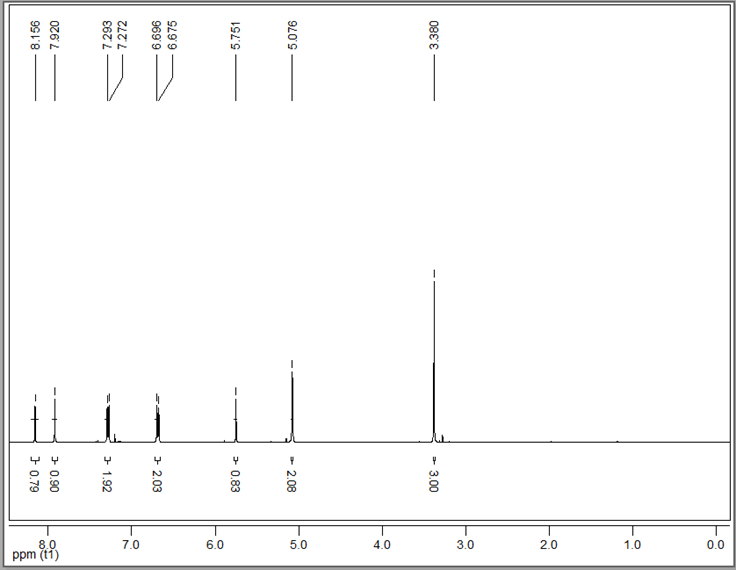


S_41_: ^1^HNMR spectrum of 4k


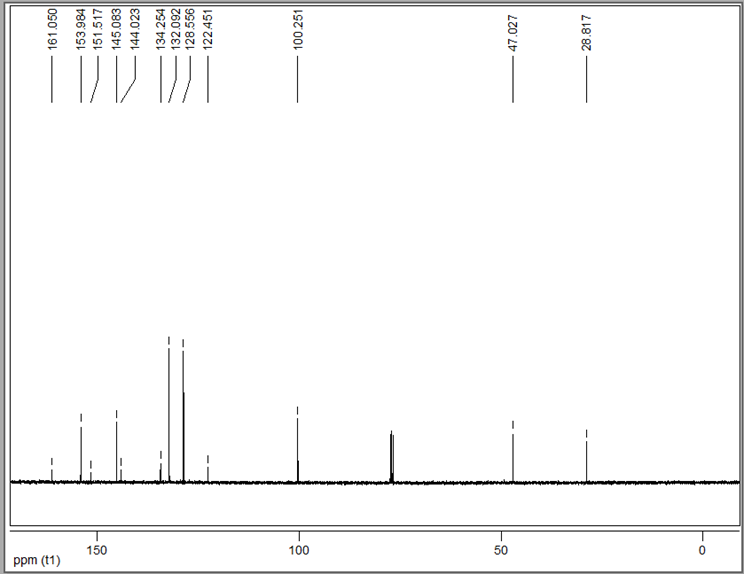

S_42_: ^13^CNMR spectrum of 4k


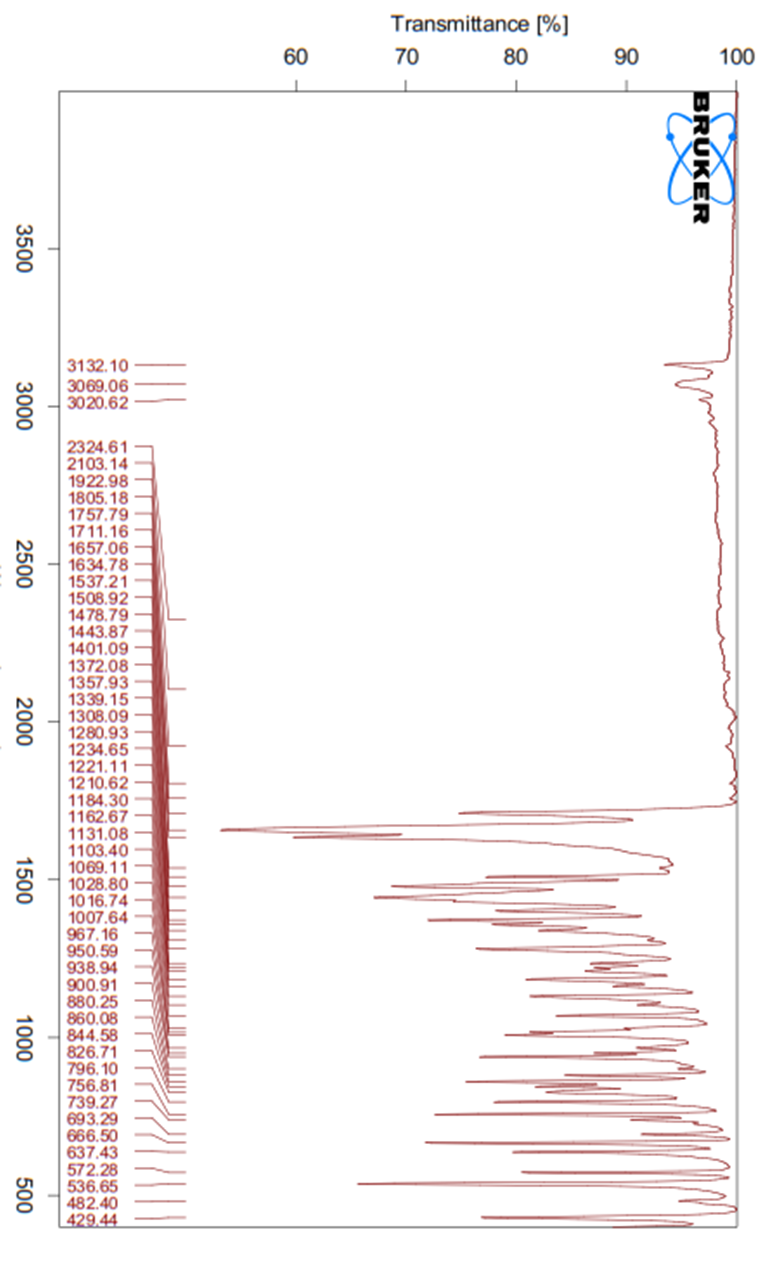

S_43_: IR spectrum of 4k


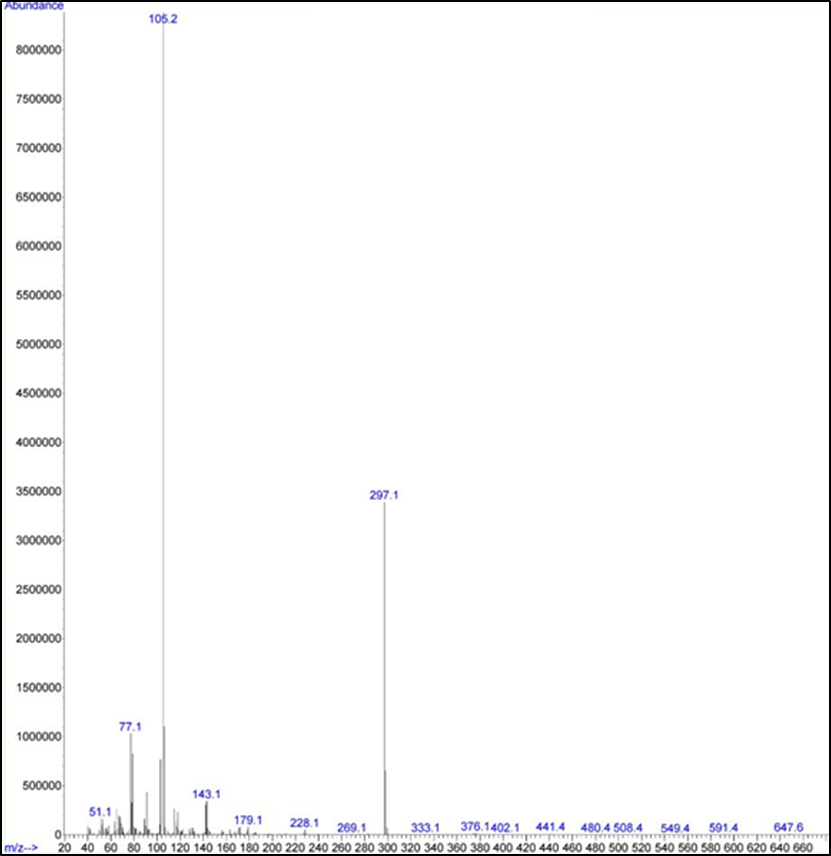


S_44_: Mass spectrum of 4l


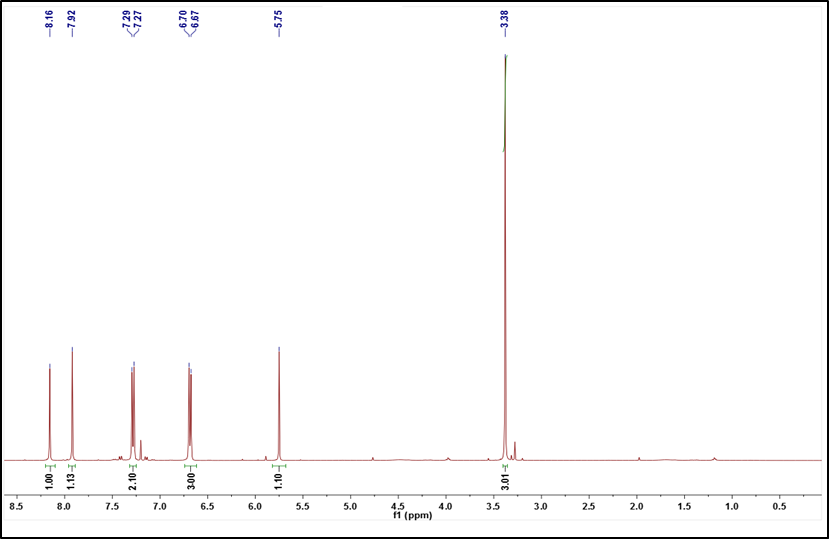


S_45_: ^1^HNMR spectrum of 4l


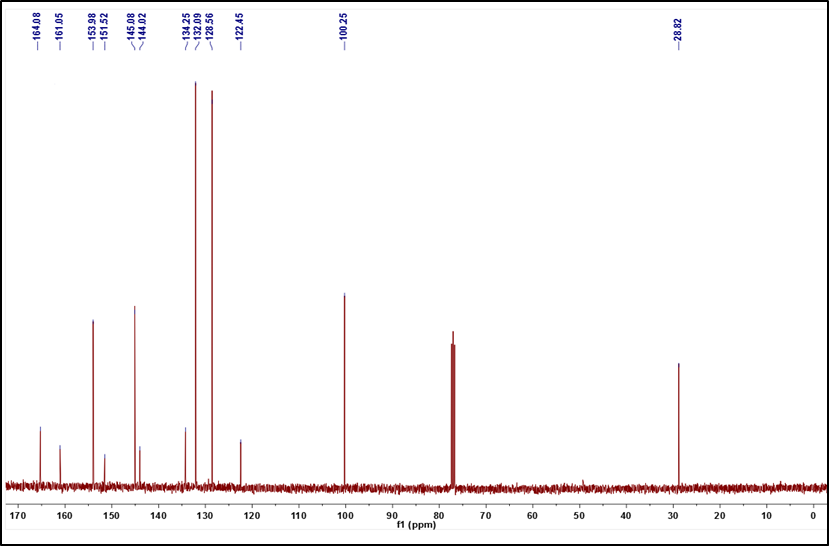


S_46_: ^13^CNMR spectrum of 4l

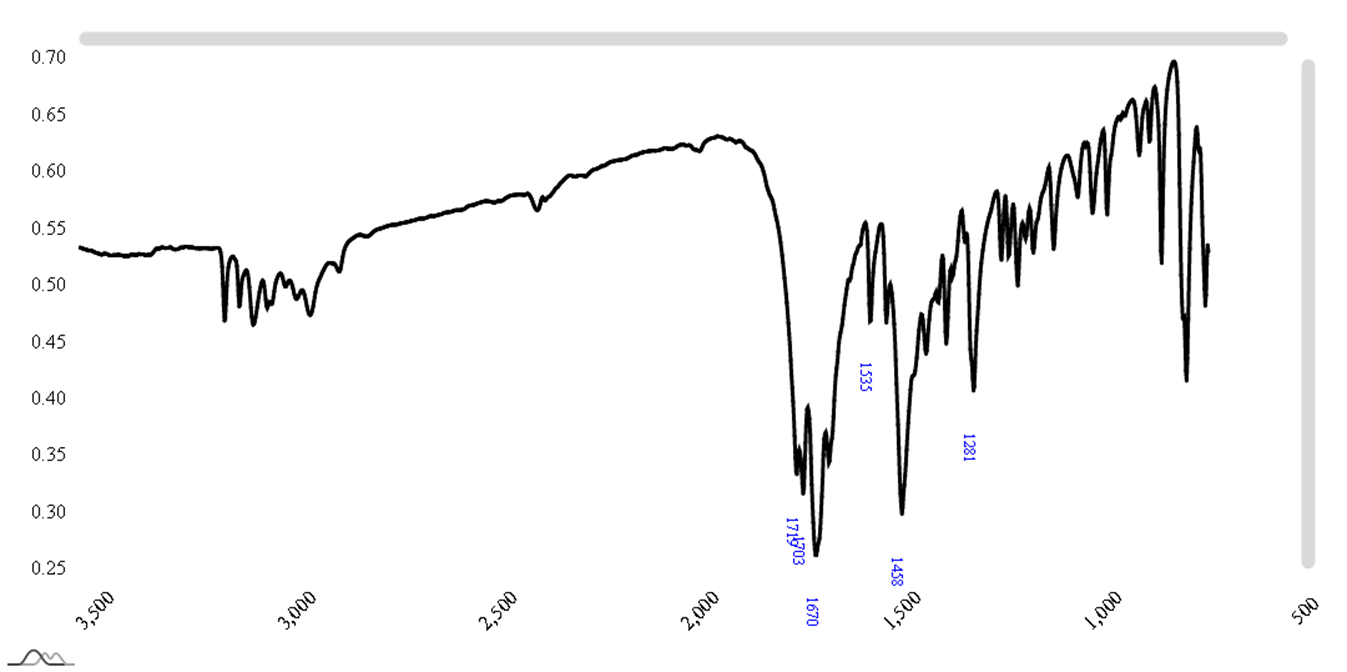


S_47_: IR spectrum of 4l

1. Corresponding author:

   Razieh Sabet, Department of Medicinal Chemistry, Faculty of Pharmacy, Shiraz University of Medical Sciences, Shiraz, Iran. Email: [sabet_r@sums.ac.ir](mailto:sabet_r@sums.ac.ir)

   a: These authors are contributed equally to this work. [↑](#footnote-ref-1)
